# Tissue fibrosis induced by radiotherapy: current understanding of the molecular mechanisms, diagnosis and therapeutic advances

**DOI:** 10.1186/s12967-023-04554-0

**Published:** 2023-10-09

**Authors:** Zuxiang Yu, Chaoyu Xu, Bin Song, Shihao Zhang, Chong Chen, Changlong Li, Shuyu Zhang

**Affiliations:** 1 Laboratory of Radiation Medicine, West China School of Basic Medical Sciences and Forensic Medicine, Sichuan University, Chengdu, 610041 China; 2 The Second Affiliated Hospital of Chengdu Medical College, China National Nuclear Corporation 416 Hospital, Chengdu, 610051 China; 3 NHC Key Laboratory of Nuclear Technology Medical Transformation (Mianyang Central Hospital), Mianyang, 621099 China; 4 Department of Gastroenterology, The First People’s Hospital of Xuzhou, Xuzhou Municipal Hospital Affiliated to Xuzhou Medical University, Xuzhou, 221200 China; 5 Department of Molecular Biology and Biochemistry, West China School of Basic Medical Sciences and Forensic Medicine, Sichuan University, Chengdu, China

**Keywords:** Ionizing radiation, Organ fibrosis, Radiation-induced fibrosis, Myofibroblast, Diagnosis, Therapy

## Abstract

Cancer remains the leading cause of death around the world. In cancer treatment, over 50% of cancer patients receive radiotherapy alone or in multimodal combinations with other therapies. One of the adverse consequences after radiation exposure is the occurrence of radiation-induced tissue fibrosis (RIF), which is characterized by the abnormal activation of myofibroblasts and the excessive accumulation of extracellular matrix. This phenotype can manifest in multiple organs, such as lung, skin, liver and kidney. In-depth studies on the mechanisms of radiation-induced fibrosis have shown that a variety of extracellular signals such as immune cells and abnormal release of cytokines, and intracellular signals such as cGAS/STING, oxidative stress response, metabolic reprogramming and proteasome pathway activation are involved in the activation of myofibroblasts. Tissue fibrosis is extremely harmful to patients' health and requires early diagnosis. In addition to traditional serum markers, histologic and imaging tests, the diagnostic potential of nuclear medicine techniques is emerging. Anti-inflammatory and antioxidant therapies are the traditional treatments for radiation-induced fibrosis. Recently, some promising therapeutic strategies have emerged, such as stem cell therapy and targeted therapies. However, incomplete knowledge of the mechanisms hinders the treatment of this disease. Here, we also highlight the potential mechanistic, diagnostic and therapeutic directions of radiation-induced fibrosis.

## Introduction

Ionizing radiation has been widely utilized in various fields such as industry, medicine and the military. In cancer treatment, more 50% cancer patients undergo radiotherapy either alone or in conjunction with other therapies. However, patients who receive tumor radiotherapy may experience severe adverse effects due to medical exposure to ionizing radiation. Although fractional radiation and other accurate techniques have been extensively applied in radiotherapy, radiation-induced tissue fibrosis remains a serious concern [1–3]. In addition, accidental and occupational exposure to ionizing radiation can also induces tissue damage. Ionizing radiation is particularly harmful to major organs throughout the body, including the heart, lung and skin, resulting in both acute and chronic damage.

Ionizing radiation has been found to cause a range of biological effects, which include oxidative damage to biological molecules like DNA, lipid, proteins and metabolites [4]. The accumulation of DNA damage and reactive oxygen species (ROS) can trigger cell death via various mechanisms, such as apoptosis, necrosis, necroptosis and ferroptosis. Additionally, ionizing radiation can lead to senescence and autophagy [5]. Ionizing radiation can have both acute and chronic biological effects, with organ fibrosis being a late symptom that occurs after exposure.

Fibrosis is the process of normal tissue components being destroyed and replaced by disordered collagen fibers and matrix. The process is typically marked by an increase in the production and deposition of extracellular matrix components (ECM), as well as a significant accumulation of myofibroblasts. Fibrotic tissue remodeling often results in organ failure and is linked to increased morbidity and mortality rates [6]. Radiation-induced tissue fibrosis causes irreversible damage to tissues and organs, exacerbating normal tissue damage around the tumor, and significantly limiting the available options for radiotherapy dose and frequency. However, the mechanism behand radiation-induced fibrosis remains elusive. Therefore, it is crucial to elucidate the regulatory mechanism underlying the development of radiation-induced tissue fibrosis to mitigate the adverse effects of radiotherapy and improve its therapeutic efficacy.

Radiation-induced fibrosis is a condition where fibroblasts remain activated and there is an excessive build-up of extracellular matrix [7]. Unfortunately, there are currently no specific or effective treatments available. We reviewed the mechanisms associated with radiation-induced fibrosis and non-radiation-induced fibrotic diseases and found that there are some commonalities between the two, such as pro-fibrotic mechanisms and cells involved in the regulation of fibrosis. Compared to radiation-induced fibrosis, non-radiation-induced fibrosis has been richly studied. The aim of this review is to summarize the mechanisms involved in radiation-induced fibrosis and non-radiation-induced fibrotic diseases, and to contribute to the study of the mechanisms of radiation-induced fibrosis. In addition, potential treatments for radiation-induced fibrosis will be discussed.

## The utilization of ionizing radiation in medicine

**Fig. 1:**
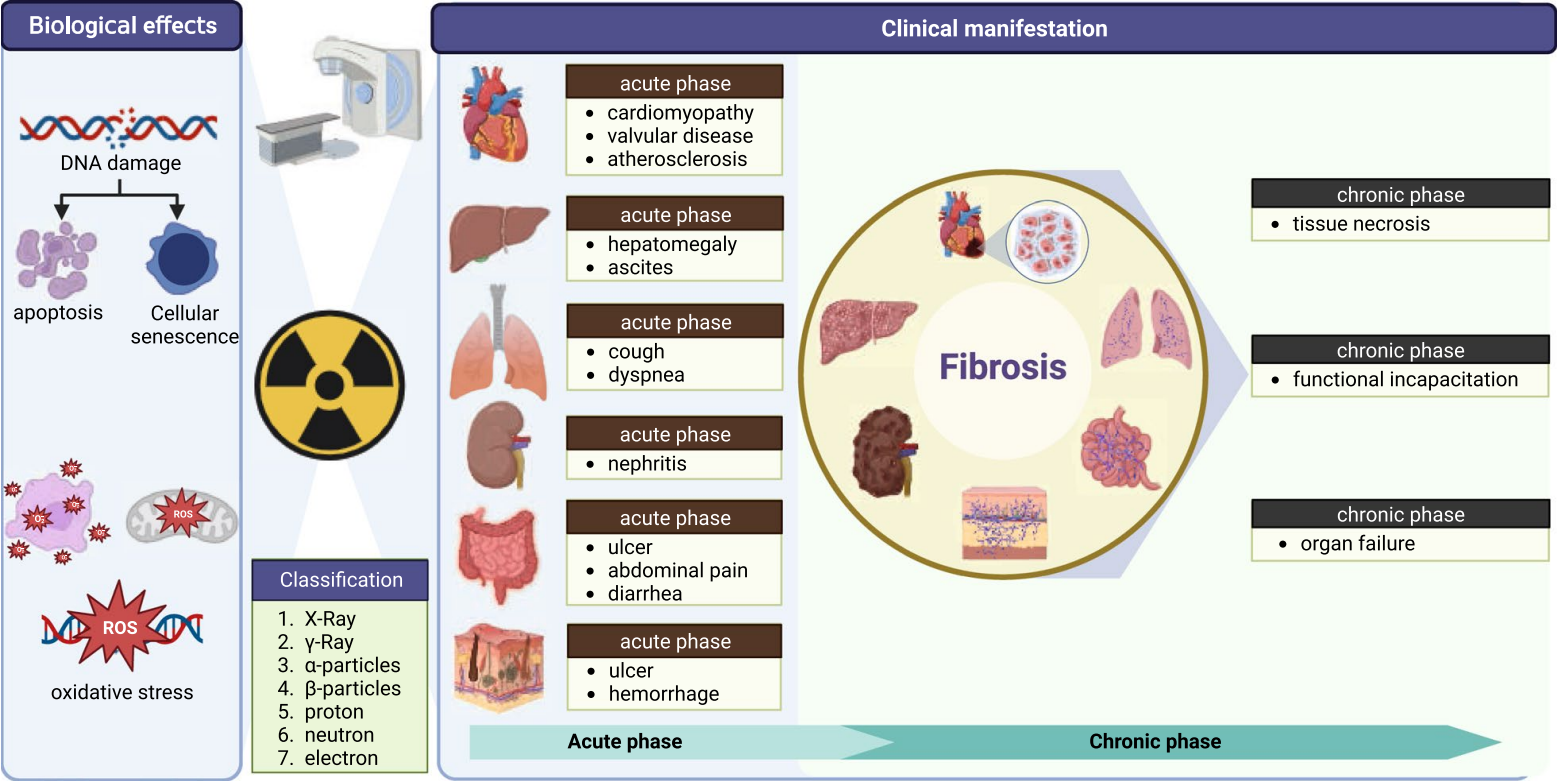
Biological effects of ionizing radiation on human normal tissues. In radiotherapy, ionizing radiation causes a variety of biological effects that result in cellular damage. Ionizing radiation causes cellular DNA breakage, leading to apoptosis and senescence. In addition, ionizing radiation causes oxidative damage due to an imbalance of oxidative stress. During cancer treatment, ionizing radiation can cause damage to the heart, liver, lung, kidney, intestines, skin and other organs. The stages of damage are mainly divided into acute and chronic fibrosis. (Figure was created with https://www.Biorender.com)

Ionizing radiation is extensively employed in medical practice for tumor radiotherapy, non-tumor radiotherapy (such as keloid) and diagnostic imaging. This type of radiation encompasses high-energy electromagnetic waves (such as X-rays and γ-rays) and high-energy particles, including ɑ-particles, β-particles, protons, neutrons and electrons. Different types of radiation have varying levels of penetration and harm to the human body. X-rays and γ-rays are highly penetrating and can cause significant damage, while ɑ-particles and β-particles are less penetrating and less harmful when exposed externally [8]. It is vital to note that ionizing radiation is not harmful to the human body below a certain threshold dose. However, exceeding this limit can cause severe tissue and organ damage or even death [9]. Exposure of the human body to ionizing radiation not only directly damages DNA but also triggers an imbalance in oxidative stress, leading to oxidative DNA damage [10] (Fig. 1). This damage initiates the DNA damage repair process in cells, which may result in apoptosis, mitotic catastrophe, and senescence if the DNA damage repair is insufficient. In addition, ionizing radiation can induce an inflammatory response, which may cause tissue damage due to cell death and local inflammation. In tumor radiotherapy, ionizing radiation is used to kill tumor cells, but it can also damage the normal tissue surrounding the tumor.

Physicians utilize ionizing radiation’s cytotoxic effects to eliminate tumor cells and enhance cancer patient’s survival rate. Nevertheless, although radiotherapy patients reap the benefits of ionizing radiation, they also experience late side effects that can significantly affect their quality of life [11] (Fig. 1). Fibrosis frequently caused by chronic inflammation and oxidative aging, is one of the most prevalent late side effects of radiotherapy [12]. In tumor radiotherapy, there are many factors that affect radiation-induced fibrosis, including the dose of radiation and the volume of tissue irradiated. Genetic factors also play an important role. For example, the increased risk of fibrosis after radiotherapy in breast cancer patients is associated with the *ATM* gene [3]. The *ATM* gene is involved in the repair of double-stranded DNA breaks. The progression of fibrosis can lead to organ failure and greatly impact the quality of life for radiotherapy patients. While ionizing radiation can cause fibrosis in almost any organ, the susceptibility varies. Patients undergoing radiotherapy for chest tumors are particularly at risk for lung and heart damage. The heart is generally known to be resilient to radiation, however, exposure to high doses of ionizing radiation can result in radiation-induced heart disease (RIHD). This condition is characterized by cardiomyopathy, valvular disease, and atherosclerosis. Furthermore, prolonged radiation exposure can lead to myocardial fibrosis, which in turn can cause arrhythmias, myocardial ischemia, and ultimately cardiomyopathy [13]. Radiation pneumonitis is a potential side effect of radiotherapy for thoracic tumors. On the other hand, radiation-induced pulmonary fibrosis is a late side effect that occurs when patients with chest tumors receive radiotherapy. Research shows that about 50% of lung cancer patients may develop radiation pneumonitis after receiving radiotherapy [14]. However, the prevalence of radiation-induced pulmonary fibrosis is only 16–28% [15]. Most patients with radiation pneumonitis recover with treatment, and only a few progresses to the fibrosis stage. Such patients will have symptoms such as decreased lung function and respiratory failure [16]. In addition to the damage to the lungs and heart, patients with abdominal tumors who undergo radiotherapy also experience harm to their liver, kidneys, and intestines which negatively impact their care. High doses of ionizing radiation can cause acute liver enlargement and ascites [17]. Additionally, it can lead to cause atrophy of the intestinal mucosa, ulceration, and symptoms of enteritis, including abdominal pain and diarrhea [18]. According to research, approximately 90% of patients experience some form of skin reaction as a result of radiotherapy, making the skin the most vulnerable area of the body to damage [19] (Fig. 1).

While the acute side effects of radiotherapy can be managed by modifying the treatment regimen, patients may still experience long-term side effects of ionizing radiation. These late effects can occur in any organ of the body and can be particularly challenging for patients (Fig. 1). Among these late effects, organ fibrosis is the most prevalent and can cause significant damage. The process of fibrosis is complex, involving factors such as inflammatory response and oxidative stress [20]. Currently, there is no standard landmark for the development of radiation-induced fibrosis. Therefore, exploring the main regulatory mechanisms involved in the development of radiation-induced fibrosis can lead to the discovery of effective treatment options for this condition.

## Mechanisms of radiation-induced fibrosis

Fibrosis is an irreversible process that can affect any organ. Since the mechanisms of fibrosis may be similar across different organs, studies focused on fibrotic disease in one organ can provide insight into the mechanisms and potential treatments for fibrosis in other organs. Fibrosis is regulated by various factors, including chronic inflammation, oxidative damage, metabolic reprogramming, and cellular senescence, working together in a synergistic manner. The activation of fibroblasts into myofibroblasts and the subsequent secretion of collagen are the primary events in tissue fibrosis. This process can be triggered by various factors such as transforming growth factors (TGFs), interleukins (ILs), and connective tissue growth factors. The pro-fibrotic microenvironment is also characterized by the presence of immune cells, including macrophages and T cells. The process involves multiple cells and cytokines making it a complex phenomenon. While research has made significant progress in understanding the mechanisms of inflammation, immune cells and cytokines that lead to fibrosis, translating these findings into clinical therapeutic approaches remains a challenge [21]. Although there are etiologic and clinical differences among fibrotic diseases, inflammatory stimulation, myofibroblast activation, and immune cell interactions are present in the development of fibrosis [22]. To better understand the mechanisms of radiation-induced fibrosis, it is necessary to first gain insight into the common factors and pathways that induce fibrosis.

### Cellular mechanisms of fibrosis

#### Myofibroblast-the manipulator of fibrosis

**Fig. 2:**
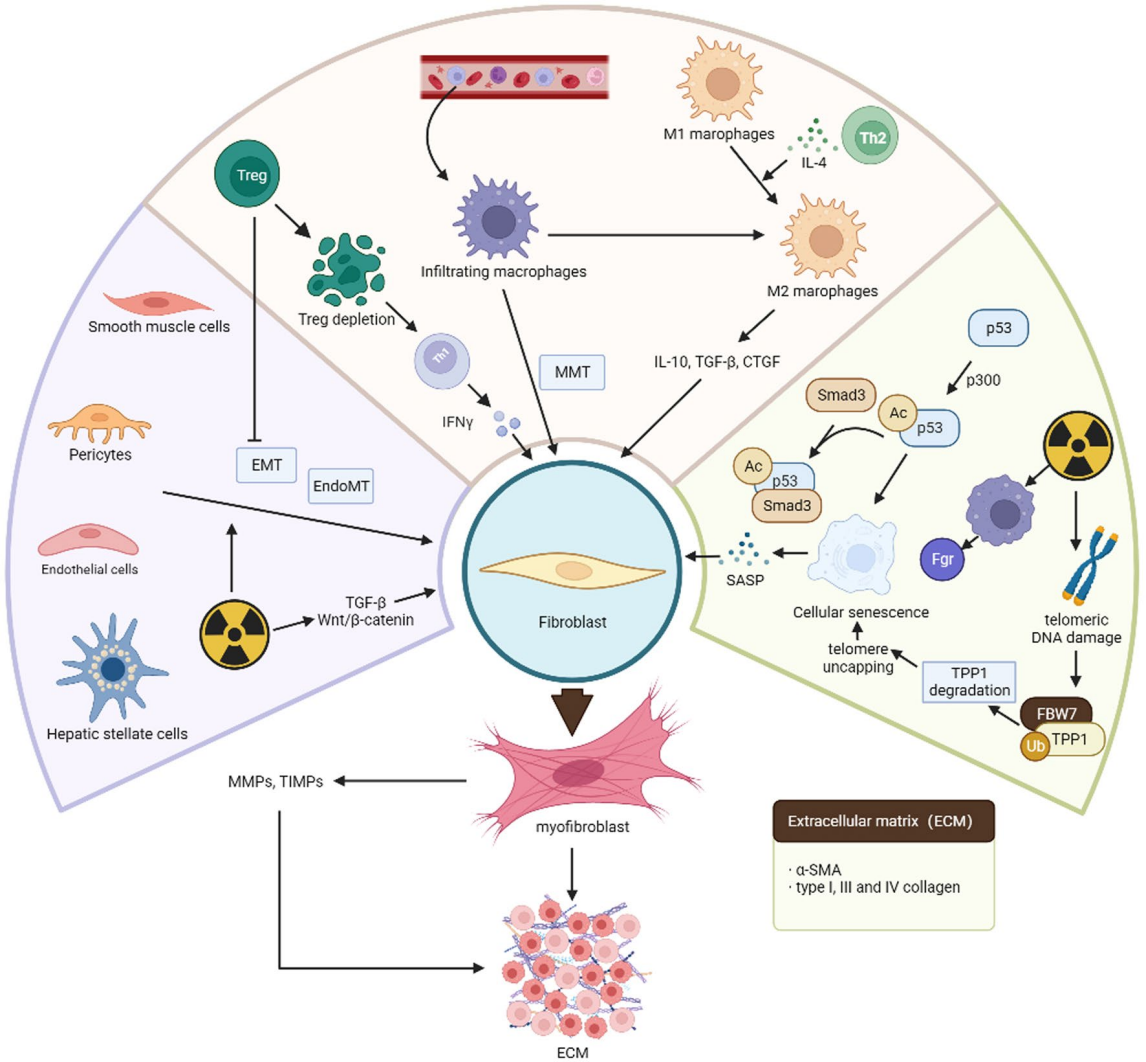
Various cells regulate the development of fibrosis. The process of fibrosis is accompanied by a variety of cellular changes. The formation of myofibroblasts is a central part of fibrosis. Cells such as fibroblasts, hepatic stellate cells and endothelial cells can be transformed into myofibroblasts and participate in the development of fibrosis. In addition, immune cells such as migrating infiltrating macrophages and T cells play a role in regulating fibrosis. Cellular senescence induced by ionizing radiation also plays an important role in fibrosis. (Figure was created with https://www.Biorender.com)

Organ fibrosis is a complex process that involves the interaction of multiple cells and cytokines. Myofibroblasts play a crucial role in this process and are the main cells responsible for extracellular matrix secretion [23] (Fig. 2). When activated myofibroblasts secrete α-smooth muscle actin (α-SMA) and type I, III and IV collagen, which are key components of the extracellular matrix [24]. Integrins link α-SMA stress fibers to collagen, resulting in high contractility that stiffens the tissue microenvironment. This can eventually lead to tissue organ sclerosis and dysfunction [25]. While high levels of α-SMA are a characteristic of myofibroblast activation, not all activated myofibroblasts exhibit high levels of α-SMA. Some α-SMA-negative myofibroblasts can also contribute to tissue fibrosis by producing stress fibers made up of β-actin [26]. Activated fibroblasts produce matrix metalloproteinases (MMPs) and tissue inhibitors of metalloproteinases (TIMPs), which play a crucial role in remodeling the extracellular matrix [27]. Multiple signaling stimuli can activate fibroblasts into myofibroblasts, with the most significant chemical signal being transforming growth factors-β (TGF-β). Additionally, changes in extracellular matrix composition can activate fibroblast transformation through cell surface receptors [28]. Recent studies have shown that myofibroblasts can be derived not only from tissue-resident fibroblasts, but also from hepatic stellate cells (HSC), endothelial cells, pericytes, and smooth muscle cells, through process such as epithelial-mesenchymal transition (EMT) and endothelial-mesenchymal transition (EndoMT) [29–32] (Fig. 2). This phenomenon explains the high abundance of myofibroblasts observed in organs affected by fibrosis, such as the heart, liver and kidney. During the process of wound healing, the persistence of myofibroblasts can lead to fibrous repair. However, it is possible for myofibroblasts to dedifferentiate into fibroblasts, which can prevent the formation of scars [33]. To our knowledge, there have been no reports on the treatment of fibrosis through the dedifferentiation of myofibroblasts.

Apart from bleomycin- and carbon tetrachloride-induced fibrosis models, radiation-induced organ fibrosis is also primary driven by myofibroblasts [34, 35]. Myofibroblast activation is promoted by ionizing radiation through the activation of various signals such as TGF-β and Wnt/β-catenin [36]. Ionizing radiation can activate fibroblasts, endothelial cells and vascular smooth muscle cells, transforming them into myofibroblasts. These cells are involved in the secretion of ECM [37] and can cause irreversible fibrosis in organs. This chronic phenotype is a result of tumor radiotherapy and severely limits the efficacy of radiotherapy. To effectively treat radiation-induced fibrosis, it is crucial to understand the source and activation mechanism of myofibroblasts, as well as the regression of these cells. This knowledge will aid in developing targeted therapies for the condition.

#### Participants in myofibroblast activation-immunocytes

##### Role of macrophage infiltration and polarization in fibrosis

In contrast to certain pro-fibrotic cells that reside in organs, infiltrating cells from nearby tissues or those that travel through the bloodstream are crucial inactivating myofibroblasts. Immune cell like macrophages and T cells are the most notable example of these cells. Prolonged inflammation triggered by ionizing radiation is a significant contributor to fibrosis. In inflamed tissues and organs, a significant number of inflammatory cells migrate and infiltrate, secreting inflammatory factors and activating myofibroblasts. Among them cells, macrophages are the most representative. Monocyte-derived macrophages are a crucial factor in radiation-induced fibrosis, as their migratory infiltration plays a significant role [38] (Fig. 2). Leve et al. found that in a CD73^−/−^ mouse model increased CD73/adenosine signaling, and hyaluronic acid promote macrophage recruitment and activate the macrophage fibrotic phenotype, which is involved in the development of radiation-induced fibrosis [39]. It is noteworthy that there are differences between tissue-infiltrating macrophages and resident macrophages. According to a study on radiation-induced lung fibrosis, it is discovered that tissue-infiltrating macrophages are capable of activating fibroblasts, while resident macrophages are not [39]. Furthermore, the removal of tissue-infiltrating macrophages has been shown to have an antifibrotic effect. The use of clinically approved colony-stimulating factor receptor-1 (CSF1R) neutralizing antibodies can also eliminate macrophage infiltration and decrease the extent of fibrosis. The role of colony-stimulating factor 1 (CSF1), which is a ligand of CSF1R, extends beyond simply recruiting macrophages. It also plays a crucial role in promoting another pro-fibrotic mechanism, namely macrophage polarization [40].

Macrophages can be categorized into two phenotypes: a pro-inflammatory M1 type and an anti-inflammatory M2 type [41]. M2 macrophages are vital in the progression of fibrosis [42]. They can secrete transforming growth factor-β (TGF-β) and IL-10 to promote the conversion of fibroblasts into myofibroblasts [43]. Additionally, they participate in fibroblast proliferation and migration by secreting connective tissue growth factor (CTGF) [44]. The conversion of macrophages into M2 is stimulated by various factors and promotes tissue fibrosis development. Additionally, M1 can also be converted to M2 phenotype when stimulated by Th2-type cytokines like IL-4. This conversion result in the secretion of pro-fibrotic factors such as TGF-β and platelet-derived growth factor (PDGF) [45].

In a mouse model of unilateral ureteral obstruction, a study found that macrophages can directly transform into myofibroblasts and contribute to fibrosis progression [46]. This process is referred to as macrophage-myofibroblast transition (MMT). However, there have been no reports of MMT in radiation-induced fibrosis models. In conclusion, tissue-infiltrating macrophages and macrophage polarization are important mechanisms of ionizing radiation-induced fibrosis. Inhibition of fibrosis-associated macrophage infiltration and modulation of macrophage polarization are novel strategies for the treatment of fibrosis.

##### Regulatory T cells modulate fibrosis

While macrophages are known to be crucial in the fibrotic process, the role of regulatory T cells (Treg) in regulating fibrosis should not be overlooked. Treg, which is a subpopulation of CD4^+^ T cells, have been found to play a critical role in radiation fibrosis [47]. Depletion of Treg leads to an increase in Th17 cells and a large amount of Th1 cytokines, such as interferon-γ secretion. This process inhibits fibroblast proliferation and reduces collagen production [48, 49] (Fig. 2). In a mouse model of Th1 cytokine deficiency, fibrosis was found to be significantly increased [50]. Treg not only regulate radiogenic fibrosis through cytokines, but they also inhibit EMT and reducing fibrosis [47]. Although the mechanism by which Treg regulate fibrosis is not fully understood, it has been found that they are involved in myofibroblast activation through extracellular signaling molecules such as TGF-β and Wnt. The mechanism of Treg regulation of fibrosis remain elusive.

#### The relationship between cellular senescence and fibrosis

Recent studies have shown that aging contributes to fibrosis [51, 52]. The key regulator of senescence namely p53 play a crucial in this process [53]. Originally identified as a tumor suppressor, p53 can activate p21 to promote cellular senescence [54]. Additionally, p53 can form a complex with Smad3 to contribute to fibrosis development [55]. In a study, it was found that p300, an acetyltransferase, plays a role in promoting senescence-associated fibrosis through the p53-Smad3 complex with the p53/p21 pathway. The acetylation of p53 induced by p300 is critical process that initiates fibrosis [56].

Numerous experiments have demonstrated that ionizing radiation induces cellular senescence, with radiation-induced macrophage senescence playing a significant role in radiation-induced fibrosis. Senescent macrophages secrete senescence-associated secretory phenotypes (SASP), such as pro-inflammatory cytokines, matrix metalloproteinases, and chemokines, that encourage fibroblasts to develop fibrogenic phenotypes [57]. Cellular senescence is characterized by its interaction with the environment. Senescent cells produce an extracellular matrix that is abundant in pro-fibrotic factors, which further promote fibroblast differentiation [58]. Additionally, senescent fibroblasts can induce senescence in neighboring fibroblasts, leading to an exacerbation of senescence-associated fibrosis [59]. In a study on irradiated mice, single-cell RNA-Seq revealed that macrophages and neutrophils undergo senescence and express elevated levels of the tyrosine kinase Fgr. This observation indicates that Fgr may play a role in development of radiation-induced fibrosis [60]. Moreover, ionizing radiation elicits a response that damages telomeric DNA. The E3 ubiquitin protein ligase FBW7 (F-box and WD40 repeat domain-containing 7) binds to and degrades telomeric protection protein 1 (TPP1), which leads to telomere uncapping and induces senescence and fibrosis [61].

However, cellular senescence is mechanism that inhibits cell proliferation. In the case of myofibroblasts, inducing fibroblast senescence compromises their function [62]. This leads to loss of myofibroblast phenotype. However, the relationship between cellular senescence and fibrosis is context-dependent and requires further investigation. Therefore, further research is necessary to fully understand the role of cellular senescence in the development of fibrosis.

### Activation of extracellular signaling molecules in myofibroblasts

#### Transforming growth factor-β (TGF-β) during tissue fibrosis

**Fig. 3:**
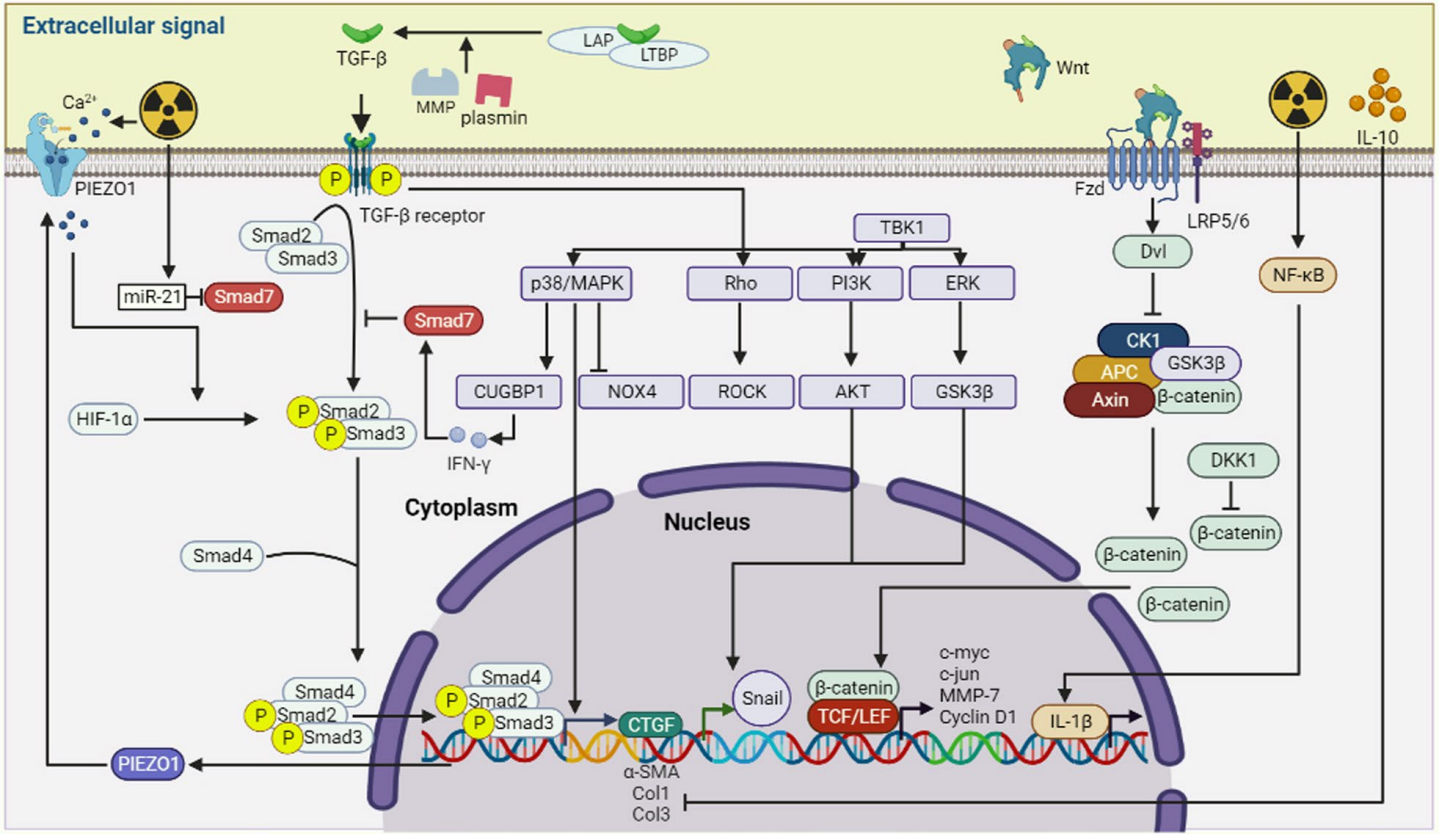
Extracellular signaling regulates fibrosis. The process of fibrosis induced by ionizing radiation is mediated by several extracellular molecules. One of the most important groups is the cytokine family, TGF-β, an important factor in the regulation of fibrosis, which can be involved in regulating the expression of fibrosis-related genes through Smad-dependent and Smad-independent pathways. In addition, extracellular Wnt signaling pathway may also be involved in regulating fibrosis gene expression by activating intracellular β-catenin signaling pathway. Cytokines such as IL-1β and IL-10 are also involved in regulating fibrosis gene expression. (Figure was created with https://www.Biorender.com)

Radiotherapy uses ionizing radiation to kills tumor cells, but it also alters cytokine levels and damages surrounding normal tissue. Among these changes, TGF-β is the most important. TGF-β induces the expression of inflammatory and fibrotic genes in normal cells, causing damage to normal tissue and interfering with the efficacy of radiotherapy [63, 64] (Fig. 3). As mentioned above, Treg cells secrete TGF-β, which plays a crucial role in regulating fibrosis [65]. TGF-β has three major isoforms, namely TGF-β1, TGF-β2, and TGF-β3. Among these, TGF-β1 is the most predominant pro-fibrotic factor and facilitates the transition of fibroblasts to myofibroblasts, thereby contributing to promotes the development of fibrosis [66].

It is currently believed that TGF-β exists mainly as a latent TGF-β/LAP/LTBP complex, which remain inactive. However, under certain pathological conditions, this complex can be cleaved by fibrinolytic enzymes, matrix metalloproteinases, and other agents, leading to the release TGF-β [67, 68]. Once released, TGF-β1 binds to type II TGF-β receptors and phosphorylates type I TGF-β receptors. TGF-β receptors of type I are activated and recruit Smad2/3, which then binds to Smad4 to form a ternary complex. This complex translocates into the nucleus, where it regulates the expression of target genes including collagen coding genes [69–71]. The activation of Smad signaling by TGF-β1 leads to the conversion of fibroblasts to myofibroblasts and promotes the expression of α-SMA and collagen [72]. The pro-fibrotic effect of TGF-β1 was validated through the use of CRISPR/Cas9 technology to knock down TGF-β1 in mice [73]. Furthermore, reduced radiation-induced fibrosis was also observed in *Smad3* KO mice [74]. During radiation-induced fibrosis, the highly activated TGF-β/Smad pathway, and reduce levels of Smad7 led to increased expression of fibrotic genes. This is especially relevant since Smad7 has been shown to be a potent inhibitor of the TGF-β/Smad pathway [75, 76]. NF-κB is a crucial inflammatory regulator that can promote the development of radiation-induced fibrosis by regulating TGF-β signaling [77].

Lipoxin A4 (LXA4), is a potent antioxidant that is produced from arachidonic acid. Studies have shown that LXA4 can attenuate liver fibrosis by targeting the TGF-β/Smad pathway [78]. Additionally, radiation has been shown to increase the expression of the LXA4 receptor (FPR2), while LXA4 itself decreases NF-κB and Smad binding element promoter activity [79]. Hypoxia-inducible factor-1α (HIF-1α) plays a crucial role in reducing radiation-induced ROS production and regulating TGF-β signaling. Its regulation is influenced by calcium signaling [80]. PIEZO1, a calcium channel, is known to increase its expression in response to ionizing radiation, resulting in an increase in inward Ca^2+^ flux. Increased levels of cytoplasmic Ca^2+^ can boost TGF-β1 signaling mediated by HIF-1α. The activated signaling pathway can release C/EBPβ from the PIEZO1 promoter by inhibiting the expression of the transcription factor C/EBPβ [81]. On the other hand, unlike the effects of LXA4 and HIF-1α, a non-coding RNA (miR-21) can interfere with antioxidants. Moreover, radiation induced the expression of miR-21, which in turn activates the TGF-β/Smad signaling pathway and suppresses the expression of Smad7. This ultimately results in the development and progression of fibrosis [82].

In addition to the canonical TGF-β/Smad pathway, other pathways such as PI3K/Akt, mitogen-activated protein kinase (MAPK), and AMP-activated protein kinase (AMPK) have been found to be activated in cardiac fibrosis models. This was discovered by constructing circRNA expression profiles and circRNA-associated ceRNA networks [83]. These pathways are part of the Smad-independent signaling pathway. Researchers found that Inhibition of p38/MAPK/Akt reduced the level of NADPH oxidase 4 (NOX4), which in turn inhibited radiation-induced myofibroblast activation [84]. The pro-fibrotic effects of MAPK have a wider impact than just bile duct ligation-induced liver fibrosis model, TGF-β signaling can cause significant activation of HSCs and stimulate high levels of CUG-binding protein 1 (CUGBP1) in HSCs through the p38/MAPK pathway. This leads to reduced expression of interferon-γ (IFN-γ). IFN-γ has been observed to induce the expression- of Smad7, which subsequently inhibits the TGF-β/Smad cascade response [85–87]. Along with various Smad-independent pathways, the PI3K/Akt is a signaling pathway associated with cell proliferation and metabolism. Inhibition of this pathway has also been demonstrated to decrease the transformation of fibroblasts into myofibroblasts [88]. EMT is a crucial factor in the activation of myofibroblasts. The activation of Smad-independent signaling pathways such as Akt and ERK/GSK3β/Snail signaling plays a significant role in this process. Studies have shown that TANK-binding kinase 1 (TBK1) can activate both signaling pathways to promote EMT, which eventually leads to organ fibrosis [89]. The Rho/Rock (Rho-associated convoluted helix kinase) signaling pathway is associated with cell differentiation and proliferation, and it is a Smad-independent signaling pathway that is closely related to radiation-induced fibrosis [90]. TGF-β, as the major signaling molecule for myofibroblast activation, has been shown to promote the development of radiation-induced fibrosis through multiple Smad-dependent and Smad-independent pathways. However, some inhibitors of the TGF-β pathway, such as Smad7, have also been shown to have anti-fibrotic effects. Current studies have focused on the crosstalk between TGF-β and other fibrosis-related pathways that regulate fibrosis, such as HIF-1α. Therefore, mastering the interactions between these pathways is of critical importance for the treatment of fibrosis.

#### The regulatory role of Wnt signaling in fibrosis

In recent years, numerous studies have demonstrated that Wnt signaling molecules play an important role in tissue fibrosis including heart, liver, kidney and other organs [91–93]. β-catenin is a component of the Wnt signaling pathway, which is important for embryonic development. Normally, β-catenin in the cytoplasm is phosphorylated by a disruption complex consist of APC, Axin, CK1α, and GSK-3β. This leads to degradation by proteasome-dependent ubiquitination, resulting in low levels of cytoplasmic β-catenin that cannot activate downstream genes [94, 95]. Dishevelled (Dsh) is phosphorylated upon binding of Wnt ligands to Frizzled (Fzd) and low-density lipoprotein receptor-associated protein 5/6 (LRP5/6) on the target cell membrane [96]. This phosphorylated Dsh then promotes the phosphorylation of GSK-3β, which in turn inhibits its activity and reduces the degradation of β-catenin [97]. The accumulation of cytoplasmic β-catenin leads to its translocation to the nucleus where it binds to nuclear T-cell factor (TCF)/lymphoid enhancer-binding factor (Lef). This binding initiates the transcription of downstream target genes such as Wnt-1-induced secreted protein (WISP1), cyclin D1, c-myc, c-jun, and MMP-7, which promote fibrosis progression [98–100] (Fig. 3). The Wnt/β-catenin signaling pathway, is a known pro-fibrotic pathway, has been shown to contribute to the progression of radiation-induced skin fibrosis [101]. MiR-17-5p, a miRNA highly expressed in hepatocellular carcinoma, activates the Wnt/β-catenin pathway and exacerbates radiation-induced liver fibrosis [102]. This highlights the significance of the Wnt/β-catenin pathway in the development of radiation-induced fibrosis.

The Dickkopf (DKK) family is a group of glycoproteins that are secreted. This family consists of five major types: DKK1-4 and Dkkl1 (soggy) [103]. DKK1 is known to be an antagonist of the Wnt/β-catenin pathway and can significantly reduce β-catenin activity, which in turn blocks the process of fibrosis [104, 105]. Additionally, human adipose-derived mesenchymal stromal cells (Ad-MSCs) can produce DKK1, which helps in reducing radiation fibrosis through the Wnt/β-catenin pathway [106]. Although the Wnt/β-catenin pathway shows great potential in the treatment of radiation-induced fibrosis, there is a lack of research in this area. Further exploration of the role of this pathway in radiation-induced fibrosis could provide valuable insights for developing effective treatment strategies.

#### Connective tissue growth factor (CCN2/CTGF), a key regulator of fibrosis

The TGF-β/Smad signaling pathway is a potent pathway that leads to fibrosis. When exposed to ionizing radiation, this pathway induces the expression of α-SMA, collagen, and other fibrosis-related factors such as CTGF [107]. CTGF (CCN2) is a member of the stromal cell protein family, is a well-studied fibrosis-related factor. It is a secreted protein that is rich in cysteine and has been shown to play a crucial role in wound healing and organ fibrosis [108, 109]. The TGF-β1/Smad/CTGF signaling pathway has been found to activate of hepatic stellate cells in a radiation-induced liver fibrosis [110]. Furthermore, TGF-β can induce the expression of CTGF through Smad-independent TGF-β signaling pathway, such as MAPK signaling and the Rho/ROCK pathway. CTGF, in turn, can activate of myofibroblasts [111–113]. In the angiotensin II-induced myocardial fibrosis model, Dorn et al. discovered that only CCN2 derived from fibroblasts can effectively stimulate the activation of cardiac fibroblasts and promote fibrosis. CCN2 from cardiomyocytes, on the other hand, does not have this function. As a result, CTGF is believed to be an autocrine regulator [114]. Although CTGF's role in fibrosis is well established, its regulation in radiation-induced fibrosis has not been confirmed. Currently, there are several strategies being explored to target CTGF for fibrosis treatment [115, 116]. However, the exact mechanism by which CTGF promotes fibrosis in the context remains unclear.

#### Role of the interleukin family members in fibrotic diseases

The inflammatory response caused by radiation is a significant mechanism for tissue damage, and cytokines from the interleukin family play a crucial role in mediating this response. Prolonged exposure to ionizing radiation can result in severe tissue fibrosis due to chronic inflammation [3]. IL-1β, secreted mainly by macrophages and dendritic cells, is the most crucial pro-inflammatory factor [117]. It binds to the IL-1β receptor on the membrane surface, stimulating the production of more IL-1β, ultimately leading to the development of fibrosis [118]. Additionally, under inflammatory conditions, IL-1β acts as a potent pro-EndMT factor, promoting the transformation of endothelial cells into activated fibroblasts and contributing to the occurrence of fibrosis [119]. Radiation can activate the NF-κB signaling pathway, leading to the production of large amounts of IL-1β. This cytokine can then recruit immune cells to the site of injury [120, 121] (Fig. 3). The CC chemokine ligand CCL2 plays a crucial role in this process. In radiation-induced lung fibrosis, IL-1β produced by lung epithelial cells, macrophages and fibroblasts stimulates the production of CCL2. The resulting increase in CCL2 levels attracts more CCR^+^ macrophages to the site of injury, exacerbating the development of radiation-induced fibrosis [122].

Interleukin 10 (IL-10) is a cytokine that has both anti-inflammatory and anti-fibrotic effects. It can inhibit the production of type I and III collagen (Col 1 and Col3), while also increasing the expression of MMP and inhibiting the transformation of fibroblasts into myofibroblasts. Additionally, IL-10 promotes the differentiation of macrophages into a regenerative phenotype, which further contributes to its antifibrotic role. These findings are supported by studies referenced as [123, 124] (Fig. 3). According to a study, the use of bone marrow mesenchymal stem cells was found to increase the number of anti-inflammatory CD163^+^ macrophages and promote the expression of IL-10 in activated macrophages in irradiated mice. This, in turn, helped in attenuating radiation-induced skin fibrosis [125]. The interleukin family consists of many members that are abundant in the inflammatory environment induced by ionizing radiation and are mainly secreted by immune cells. Different interleukins can exert pro- and anti-fibrotic effects. Their specific regulatory fibrotic effects need to be further investigated.

### Intracellular changes in fibrosis-associated cells

#### Progression of fibrosis due to oxidative stress imbalance

**Fig. 4:**
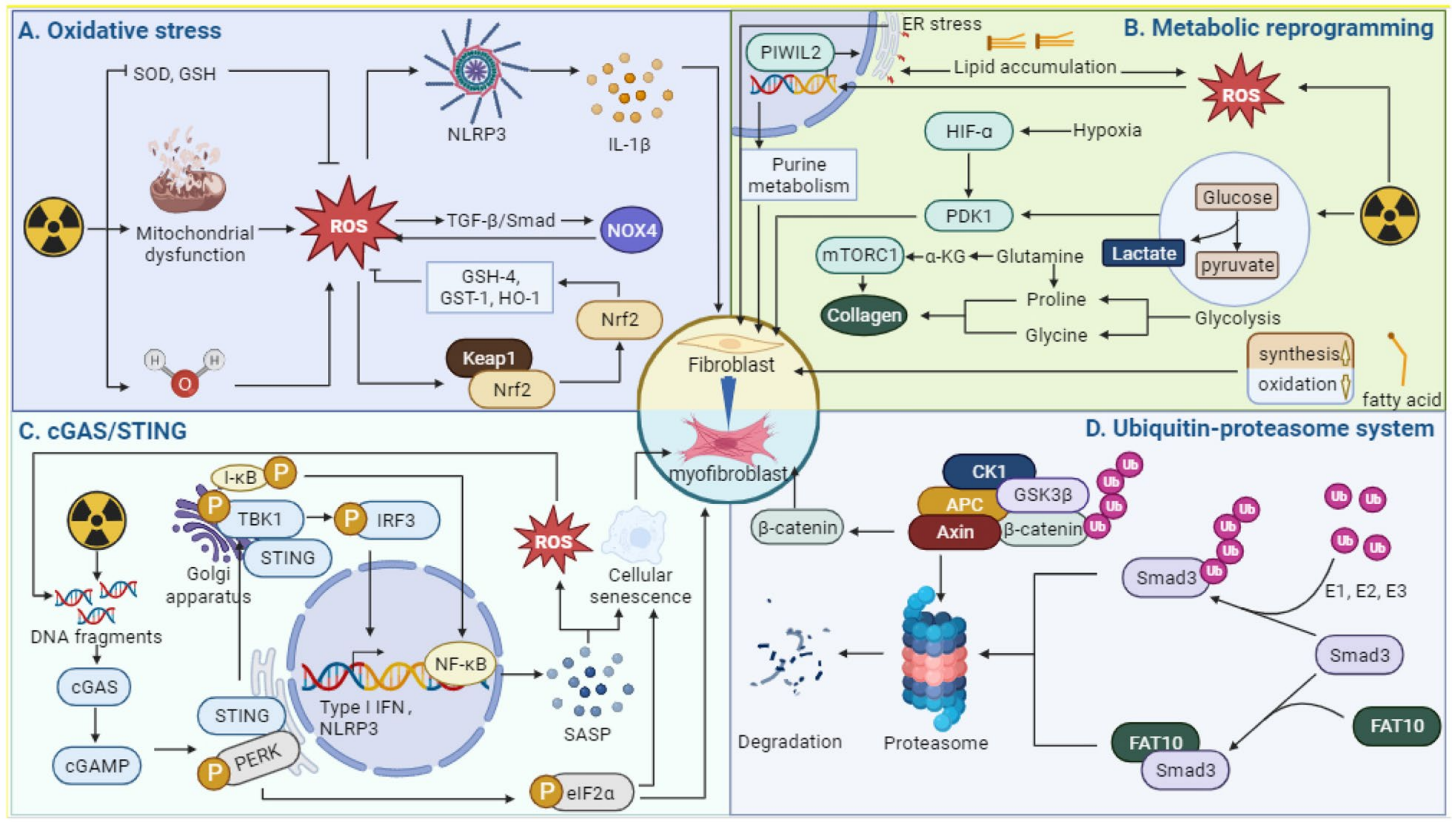
Changes in intracellular components in fibrosis. **A** Ionizing radiation promotes fibrosis by generating ROS and disrupting the activity of antioxidant enzymes such as SOD and GSH. **B** Ionizing radiation can alter intracellular glucose metabolism, amino acid metabolism, and lipid metabolism pathways through metabolic reprogramming, thereby promoting fibrosis. **C** DNA damage caused by ionizing radiation can result in large amounts of DNA fragmentation. These free DNA fragments activate the cGAS/STING pathway and promote fibrosis. **D** The ubiquitin–proteasome pathway and the FAT-10 proteasome pathway mediate the degradation of key fibrosis signaling molecules such as Smad3 and β-catenin, thereby promoting fibrosis. (Figure was created with https://www.Biorender.com)

Ionizing radiation is known to cause cellular damage through oxidative stress (Fig. 4). This occurs when free radical production becomes abnormal and damages the antioxidant system. Free radicals are classified into two types: reactive oxygen species (ROS) and reactive nitrogen species (RNS). The production of ROS from water in the cytoplasm, specifically hydroxyl radicals (·OH) and superoxide anions (O^2−^·), is induced by ionizing radiation [7]. In addition, ionizing radiation can disrupt the mitochondrial electron transport chain as well as superoxide dismutase and glutathione antioxidant enzymes, leading to a significant accumulation of ROS [126].

Mitochondria are organelles responsible for generating energy within cells and are closely involved in regulating oxidative stress. Several studies have shown that dysfunction of mitochondria and imbalanced oxidative stress can contribute to the development of organ fibrosis. Specifically, mitochondrial dysfunction can lead to the production of reactive oxygen species (mtROS), which promote fibrosis, while oxidative stress imbalance can result in mitochondrial dysfunction, damage to mitochondrial DNA, and other factors that promote fibrosis [127, 128]. ROS plays a significant role in promoting myofibroblast activation by increasing the expression of NOX4 through the TGF-β/Smad pathway [129]. Additionally, ROS can also activate NOD-like receptor protein 3 (NLRP3) and its downstream factor IL-1β [130]. This highlights the crucial role of ROS in the activation of fibrosis. The upregulation of DPYSL4, a mitochondrial super-complex-related gene, in post-illumination lung epithelial cells stimulates the production of ATP and NADPH, leading to a reduction in pro-fibrotic ROS production [130]. Mitochondrial enzymes also play a crucial role in regulating ROS levels. In a mouse model of CCI_4_-induced liver fibrosis, the enzyme p66Shc was discovered to promote the production of ROS, which activates inflammatory vesicles NLRP3 and is involved in the progression of fibrosis. This was reported in a study with reference [131].

Nuclear factor erythroid-2-related factor 2 (Nrf2) regulates intracellular antioxidant and plays a crucial role in reducing ionizing radiation-induced oxidative damage. In normal conditions, Nrf2 remains inactive as it binds to cytoplasmic Kelch-like ECH-related protein 1 (Keap1). However, when there is an imbalance in intracellular oxidative stress, the Nrf2 pathway gets activated, promoting the expression of downstream antioxidant proteins such as glutathione peroxidase-4, GST-1, and HO-1. These proteins work together to reduce ROS levels [132, 133]. Lowering ROS levels can reduce TGF-β signaling and consequently decrease radiographic fibrosis [134]. However, in Nrf^2−/−^ mice, collagen secretion is increased due to activated pro-fibrosis signaling [135]. Additionally, the aberrant expression of receptor-interacting protein kinase-3 (RIPK3), a factor associated with necroptosis, can lead to mitochondrial dysfunction [136]. Studies have demonstrated that Nrf2 can impede RIPK3-induced mitochondrial dysfunction, which in turn suppresses ROS the production of ROS and accumulation of collagen [135]. In addition, zinc is a trace element with antioxidant properties and a crucial component of both superoxide dismutase (SOD) and MMPs. A deficiency in zinc results in reduced levels of ROS, which can contribute to the progression of fibrosis [137, 138]. Mitochondrial damage is a significant contributor to the buildup of ROS within the cell. This damage can be mitigated by the cellular antioxidant defense mechanism known as mitochondrial autophagy [139].

Although zinc and mitochondrial autophagy have been extensively studied in models of fibrosis induced by non-radiological factors, their role in in protecting against radiation-induced fibrosis remains unclear. Therefore, further research is required to investigate the mechanisms of cellular antioxidant damage protection in such cases.

#### cGAS/STING pathway-identifier of DNA damage

Ionizing radiation and the resulting oxidative damage can cause breaks in both nuclear and mitochondrial DNA, ultimately leading to cell death. This process results in the scattering of numerous DNA fragments throughout the cytoplasm. However, the cell has a mechanism to address this DNA fragmentation called the Stimulator of Interferon Genes (STING). STING is a crucial signaling molecule for intrinsic immunity and is primary located in the endoplasmic reticulum. When cells are exposed to Ionizing radiation, cellular and mitochondrial DNA damage occurs, leading to the release of free DNA into the cytoplasm. This free DNA is then detected by the cytoplasmic dsDNA recognizer cyclic guanosine adenosine phosphate synthase (cGAS), which catalyzes cyclic dinucleotide 2′3' cyclic GMP-AMP (cGAMP). The cGAS/STING pathway play a crucial role in radiation-induced injury by activating downstream STING, which then translocate to the Golgi apparatus and activates TBK1. The activation of TBK1 lead to the activation of interferon regulatory factor 3 (IRF3), which induces the expression of type I interferon and pro-inflammatory cytokines [140, 141]. Thus, the cGAS/STING pathway plays an important role in radiation-induced injury (Fig. 4). The involvement of STING pathway in DNA damage caused by ionizing radiation-induced fibrosis remains unclear.

A recent study showed that the cGAS/STING pathway is enriched in radiation-induced fibrosis [142]. Furthermore, the role of the STING pathway in liver, kidney and lung fibrosis has also been demonstrated [143–145]. Conversely, drug inhibition of STING/TBK1 signaling has been shown to reduce the conversion of bone marrow fibroblasts and macrophages into myofibroblasts [146], thus presenting is a promising pathway for targeted fibrosis therapy. Ferroptosis is a type of cell death that is dependent on iron and causes oxidative DNA damage. The damage can activate the STING pathway and ultimately lead to fibrosis [147]. Like the effects of ionizing radiation. In a study by Wang et al. found that mtDNA released from mitochondrial damage can promote fibrosis development by activating NLRP3 through cGAS/STING signaling. The process of endoplasmic reticulum stress can be regulated by the ER stress signal X-box binding protein 1 (XBP1) and its downstream factor BCL2/adenovirus E1B interaction protein 3 (BNIP3) [148]. It is necessary to note that this type of stress often occurs during the production of large amounts of extracellular matrix. Previous studies have shown that STING is an important regulator of ER stress and that it can lead to fibrosis [149]. Notably, the researchers observed elevated expression levels of three proteins, namely phosphorylated inositol-requiring kinase (p-IRE-1α), phosphorylated eukaryotic initiation factor 2α (p-eIF2α), and phosphorylated protein kinase RNA-like endoplasmic reticulum kinase (p-PERK), in a mouse model of cardiac fibrosis. This finding suggests that these three proteins may play a crucial role in the development of this pro-fibrotic process. IRE-1α is the upstream molecule of the endoplasmic reticulum stress signal XBP1 and plays a key role in its activation [148]. In contrast, two proteins, eIF2α and PERK, are associated with a non-classical STING pathway. In this pathway, the STING signal can directly activate PERK at the endoplasmic reticulum, which in turn phosphorylate eIF2α, regulating the selective expression of certain proteins. This differs from the conventional STING-TBK1-IRF3 pathway. The cGAS/STING pathway is crucial in regulating cellular senescence and fibrosis [150]. As highlight in previous research on the importance of cellular senescence in fibrosis. Notably, our findings indicate that this pathway serves as a key regulator of senescence [151]. Previous research used Oroxylin A to inhibit the production of methionine metabolites, specifically the methyl donor S-adenosylmethionine, leading to a reduction in DNA methyltransferase (DNMT)-mediated methylation of the cGAS gene [152]. Additionally, activated STING play a role in promoting NF-κB/I-κB activation, which further contributes to STING pathway activation and exacerbates ROS-induced oxidative DNA damage [152]. This promotes cellular senescence, but ultimately presents an anti-fibrotic function. This suggests that the relationship between senescence and fibrosis remains to be discussed.

Surprisingly, the relationship between the cGAS/STING pathway and fibrosis is controversial. While pharmacological activation of STING signaling has been found to play an anti-fibrotic role in mouse liver and kidney [153]. However, overexpression of STING attenuates the development of fibrosis by inhibiting autophagy [154]. In summary, the production of cytoplasmic free DNA is an important feature of ionizing radiation-induced damage. And the cGAS/STING pathway plays an important role in radiation-induced fibrosis because of its role in recognizing DNA fragments. Recent studies have identified some of the downstream effects of STING, such as cellular senescence and NLRP3 activation. However, to develop anti-fibrotic drugs targeting the STING pathway, further dissection of the fibrosis-related effects downstream of the STING pathway is needed.

#### Metabolic reprogramming in fibrosis

The process of fibrosis results in the production of a significant amount of matrix, including ɑ-SMA and collagen. This is generally considered an anabolic process that consumes energy, leading to an increase in energy metabolism and anabolism [155]. Mitochondria plays a crucial role in this process. The organism undergoes metabolic reprogramming as an adaptive process in response to external stimuli (Fig. 4). This process involves various metabolic pathways, including gluconeogenesis, amino acid metabolism and lipid metabolism. While the process of energy changes is complex, a study on cardiac fibrosis, using a proteomic approach found that ionizing radiation has an impact on cardiac energy metabolism in mice, ultimately affecting the onset of cardiac fibrosis [156]. Due to energy for cell proliferation, repair, synthesis and secretion are primary produced through aerobic mitochondrial respiration. Pro-fibrotic factors, including TGF-β and ionizing radiation, have been shown to reprogram cellular metabolism and promote myofibroblast formation through the enhancement of aerobic glycolysis [157–159]. This process leads to a significant increase in pyruvate dehydrogenase 1 (PDK1) during glycolysis. HIF-1α is a key factor in regulating anaerobic glycolysis to adapt to hypoxic environments and induces myofibroblast differentiation by targeting PDK1 [160]. The end product of glycolysis (lactic acid) has been identified as a promoter of collagen hydroxylation through proline hydroxylase activity [161]. Glucose transporter protein 1 (GLUT1), which is the most abundant glucose transporter protein in mammals, plays a crucial role in glucose transport. In a study conducted by Cho et al., it was discovered that GLUT1-dependent glycolysis could promote pulmonary fibrosis induced by bleomycin [162]. Moreover, the pro-fibrotic effect of GLUT1-dependent glycolysis was found to be closely linked with the inflammatory sensor AIM2 [163]. Additionally, a separate study discovered that purine metabolism, in addition to gluconeogenesis, plays a role in the advancement of fibrosis [164]. As previously mentioned, Nrf2, is a crucial regulator of radiogenic fibrosis. It can enhance the expression of Piwi-like RNA-mediated gene silencing 2, which in turn activates purine metabolism to repair the damage caused by ionizing radiation [165].

In addition to promoting fibrosis, glycolysis can also provide essential amino acids such as proline and glycine for collagen synthesis [166]. The serine-glycine pathway can convert glycerol-3-phosphate, a glycolytic intermediate, to produce a significant amount of glycine, which is the primary component of collagen synthesis [166]. And proline can be converted from glutamine [167]. Glutamine catabolism is a crucial topic as it serves as a biosynthetic precursor and energy donor. Its catabolism is closely linked to the progression of fibrosis [168]. The conversion of glutamine to glutamate is catalyzed by glutaminase in this process. Furthermore, α-ketoglutarate can be produced from glutamine and this compound can activate the downstream rapamycin complex 1 (mTORC1), which in turn promotes collagen synthesis as indicated by reference [169].

Apart from sugar and amino acid metabolism, lipid metabolism also plays a significant role in the fibrotic response. Generally, fibrosis leads to a reduction in fatty acid oxidation (FAO) and arise in fatty acid synthesis [170]. In a mouse model of unilateral ureteral obstruction, the activation of signal transducer and activator of transcription 6 (STAT6) inhibits FAO, leading to lipid accumulation and the development of fibrosis [170]. After knocking down STAT6, the effect was no longer present, indicating its dependence on STAT6. Additionally, radiation-induced skin fibrosis was found to be exacerbated by the impairment of FAO [159]. Furthermore, increased fatty acid synthesis has also been shown to have a pro-fibrotic effect [171]. However, the pro-fibrotic mechanisms of lipid metabolism disorders are not limited to these findings and require further investigation [172]. The uptake of triglyceride-rich very low-density lipoproteins into the cell is promoted, which leads to excessive intracellular lipid accumulation. This accumulation of lipids causes the production of ROS and endoplasmic reticulum stress, ultimately resulting in fibrosis. This process involves the activation of the type 2 immune response mediated by group 2 innate lymphoid cells of the pancreas, which is triggered by IL-33 secreted by pancreatic stellate cells [173]. Fibrosis is essentially an anabolic process in which large amounts of extracellular matrix are synthesized. Alterations in glucose metabolism, amino acid metabolism, and lipid metabolism have been shown to play an important role in radiographic fibrosis. Alterations in cellular metabolism not only provide energy for the initiation of fibrosis, but also provide some essential raw materials for fibrosis. Thus, alterations in metabolic pathways are an important mechanism of ionizing radiation-induced fibrosis.

#### Ubiquitin–proteasome system-potential regulators of fibrotic pathways

The progression of fibrosis involves various signaling molecules, and their abnormal expression or degradation is crucial in the body's resistance to fibrosis. Several studies have demonstrated the involvement of the ubiquitin–proteasome system (UPS), a protein degradation pathway, in the progression of fibrosis [174–176] (Fig. 4). Application of the proteasome inhibitor Delanzomib in a mouse model of renal fibrosis showed potential anti-fibrotic therapeutic effects [177]. Another study conducted on radiation-induced skin injury in rats revealed that the UPS is involved in the induction of radiation-induced fibrosis [178]. The UPS is mainly composed of ubiquitin, ubiquitin-activating enzyme E1, ubiquitin-binding enzyme E2, ubiquitin-protein ligase E3, 26S proteasome and ubiquitin-dissociating enzyme (DUB) [179]. The UPS pathway consists of two main steps: first, E1, E2 and E3 catalyze the ubiquitination of target protein. Second, the ubiquitinated protein is degraded by the 26S proteasome [180]. This pathway plays an important role in maintaining the normal cellular state by removing abnormal and damaged proteins. The 26S proteasome serves as the site of protein degradation in the UPS. The pro-fibrotic signal TGF-β can induce myofibroblast transformation by activating 26S proteasome. This activation is associated with the regulatory subunit Rpn6 [181].

The ubiquitin–proteasome system has been found to affect the onset of fibrosis by interfering with various pro-fibrotic pathways such as TGF-β/Smad, Wnt/β-catenin, and the transcriptional co-activator Yes-associated protein (YAP). A recent study discovered that the degradation of Smad3 through the proteasome by human leukocyte antigen F-associated transcript 10 (FAT10) could impede the development of fibrosis [182]. This process described does not involve protein ubiquitination and therefore cannot be referred to as UPS. It has been given a new name: the FAT10-proteasome system (FPS) (Fig. 4). While UPS has been shown to be effective in inhibiting fibrosis through Smad3 degradation, other means such as the regulation of tissue fibrosis [183]. Alkylation repair homolog 5 (ALKBH5) is an important protein that regulates tissue fibrosis. In a mouse model of pulmonary fibrosis exposed to PM2.5 [184], reduction of ALKBH5 activated the pro-fibrotic signaling pathway YAP, which subsequently increased the expression of P4HA2. P4HA2 is a crucial enzyme for collagen formation [185]. Alongside E3 ubiquitin ligases, DUB, another member of the UPS, also contributes significantly to the progression of fibrosis. DUB, a regulatory protein, plays a vital role in the regulation of fibrotic signaling pathways by preventing the degradation of target proteins via deubiquitinating [186]. One such DUB, ubiquitin-specific protein hydrolase 2 (USP2, a DUB) can deubiquitinate β-catenin, which leads to increased β-catenin entry into the nucleus and promoting the expression of fibrotic genes [187]. Therefore, utilizing the ability of the UPS to degrade target proteins may offer a promising research direction for the treatment of fibrosis.

## Mechanism of radiation-induced organ fibrosis

**Fig. 5:**
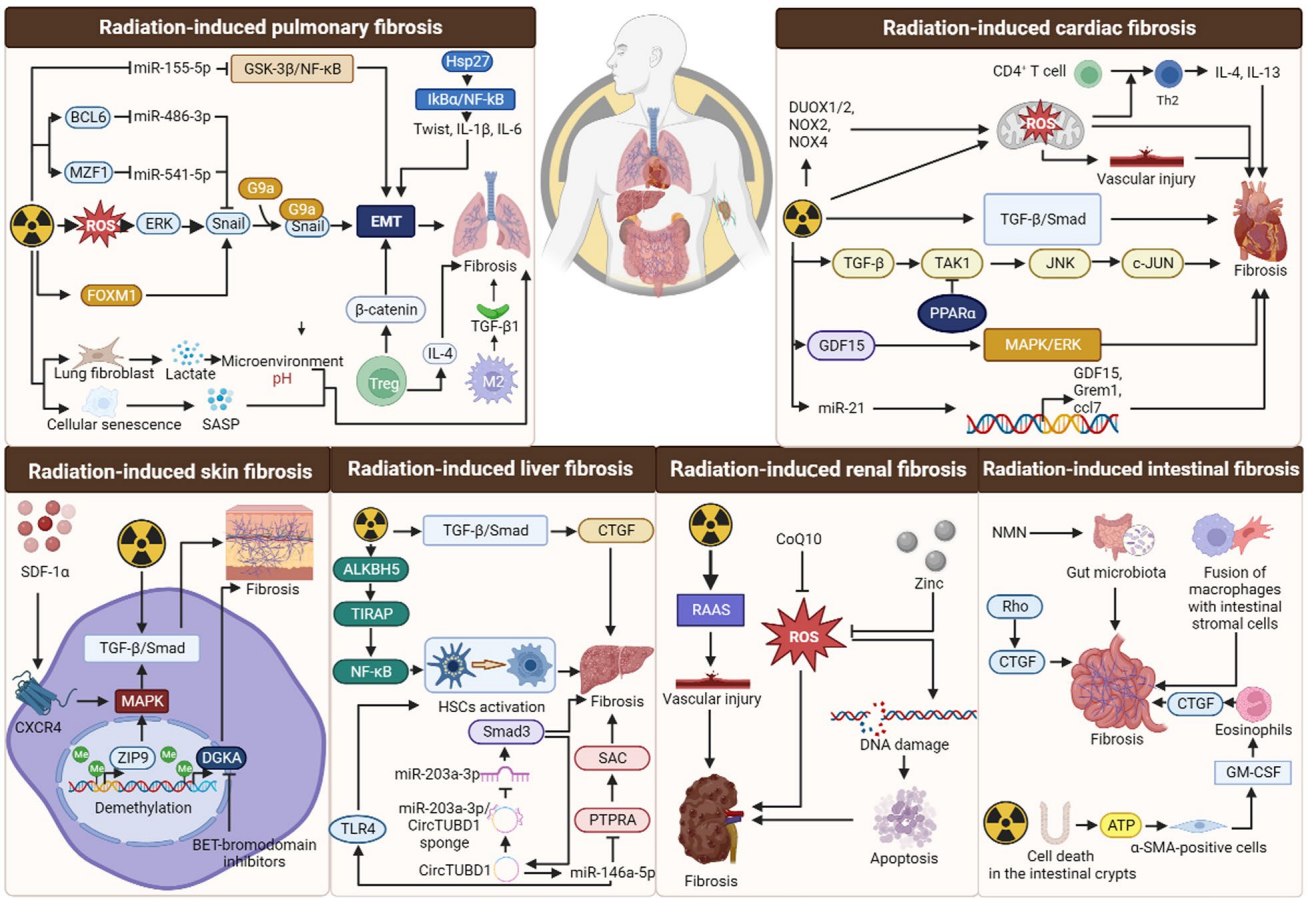
Mechanisms of radiation-induced fibrosis in various tissues. Ionizing radiation causes fibrosis in the lungs, heart, skin, liver, kidney and intestine by modualting a variety of molecules involved in cytokine signaling, collagen biosynthesis, oxidative stress response and epigenetic regulation. (Figure was created with https://www.Biorender.com)

Long-term exposure to ionizing radiation can result in fibrosis of various organs such as the heart, liver, lungs and skin. The degree of radiation-induced organ fibrosis is influenced by the radiation dose, the mode of exposure, and individual genetic susceptibility [188]. The radiosensitivity of different organs varies due to a variety of factors. And sensitive organs are more prone to developing fibrotic changes with prolonged exposure to ionizing radiation. Radiation-induced fibrosis leads to both structural changes and functional failure of organs. While the specific organs affected by radiation damage may vary, there are similarities in the cellular and molecular mechanisms underlying radiogenic fibrosis across different organs [189] (Fig. 5).

### Radiation-induced pulmonary fibrosis

Radiation-induced pulmonary fibrosis is a frequently observed clinical complication in patients receiving radiotherapy for thoracic cancer. This condition usually manifests several months after radiation treatment. The process of epithelial-mesenchymal transition triggered by ionizing radiation is a key mechanism responsible for the development of radiation-induced pulmonary fibrosis [190]. Ionizing radiation triggers the generation of significant levels of ROS, which in turn modulates the formation of complexes between Snail and G9a via the ROS/ERK pathway. This process leads to an increase in H3K9 methylation at the E-cadherin promoter, ultimately resulting in the inhibition of E-cadherin expression [191]. The inhibition of EMT and reduction of fibrosis are the ultimate outcomes. The promotion of fibrosis in ionizing radiation-induced EMT is facilitated by Heat shock protein 27 which promotes NF-kB release through the IkBα-NFkB pathway and inducing pro-fibrotic factors such as Twist, IL-1β and IL-6 [192]. Furthermore, the upregulation of transcription factor forkhead box M1 (FOXM1) is observed in alveolar epithelial cells after irradiation. FOXM1 activates Snail expression by binding to its promoter, promote, which in turn promotes EMT and increases the degree of lung fibrosis after irradiation [193]. Recent studies on non-coding RNAs have revealed that several miRNAs are involved in regulating EMT and contribute to the development of radiation-induced pulmonary fibrosis. For example, ionizing radiation can activate myeloid zinc finger protein 1 (MZF1) and activated MZF1 can inhibit the production of miR-541-5p precursors and thus reduce miR-541-5p levels. This also leads to the expression of Slug (Snail2), a key gene in EMT, which allows the exacerbation of pulmonary fibrosis after radiation exposure [194]. Moreover, ionizing radiation stimulation activates B-cell lymphoma 6 protein (BCL6) from the BTB/POZ zinc finger protein family, which inhibits the expression of downstream miR-486-3p and increases the expression of Snail, thus promoting EMT [195]. According to research, radiation exposure leads to a reduction in miR-155-5p levels in alveolar epithelial cells. This, in turn, activates GSK-3β, which is the target of miR-155-5p, and facilitates the phosphorylation of the NF-κB subunit p65. This activation of the GSK-3β/NF-κB pathway ultimately results in pulmonary EMT and the development of pulmonary fibrosis [196]. There are numerous other mechanisms implicated in radiation-induced pulmonary fibrosis. Sphingosine-1-phosphate receptor 3 (SIPR3) is a vital pro-fibrotic factor that can lead to radiation-induced pulmonary fibrosis. It does so by activating TGF-β1 signaling and increasing the expression of Snail. However, this process can be reversed by inhibiting miR-495-3p (target gene for SIPR3). This funding presents a new approach to treating radiation-induced pulmonary fibrosis [197].

In addition to miRNAs, Treg and macrophages are crucial factors in the regulation of radiation-induced pulmonary fibrosis. Treg can worsen radiation-induced pulmonary fibrosis by promoting β-catenin-mediated EMT, which severely restricts the use of radiotherapy [47]. Treg cells have been shown to produce Th2 cytokines, including IL-4, which have potent pro-fibrotic effects [198]. It has been suggested that the regulation of cytokines by Treg cells may be dependent on the activation of M2-type macrophages [199]. After radiation exposure, the lung is infiltrated by a significant number of M2 macrophages These macrophages produce high levels of TGF-β, which in turn promotes the mesenchymal transformation of lung epithelial cells [197, 200]. Phosphorylation of STAT3 mediates inhibition of Rho-associated convoluted helix kinase (ROCK), which in turn inhibits the conversion of macrophages to M2 and attenuates the extent of post-irradiation lung fibrosis [201].

Ionizing radiation has been shown to induce cellular senescence and secrete tyrosine kinase Fgr to promote the development of pulmonary fibrosis [60]. Senescent macrophages secrete SASP, such as tumor necrosis factor α, IL-1α, which are crucial in the development of pulmonary fibrosis [57]. Similarly, when type II alveolar cells are exposed to ionizing radiation, they undergo senescence and secrete a significant amount of SASP in response to IGF-1 signaling. This SASP induces IL-13, which is a powerful M2-inducing factor that promotes the development of pulmonary fibrosis [202].

ROS production is the canonical mechanism of tissue damage caused by ionizing radiation. Several studies have confirmed the role of ROS in the progression of radiation-induced fibrosis. The production of ROS triggered by radiation activates downstream NLRP3 inflammatory vesicles, resulting in increased production of IL-β by lung epithelial cells, which directly affects the transformation of fibroblasts into myofibroblasts. At the same time, ionizing radiation also activates the p53-mediated mitochondrial super-complex-related gene DPYSL4, which increases intracellular ATP levels and reduces ROS levels, thereby inhibiting NLRP3 activation [130, 203]. However, the classical pro-fibrotic signal, TGF-β, is also essential to investigate the mechanism of radiation-induced lung fibrosis. It is well known that the microenvironment in which cells are located is also a risk factor for fibrosis. Studies have shown that radiation can induce lung fibroblasts to secrete high amounts of lactate, leading to acidification of the extracellular environment and subsequent activation of pro-fibrotic TGF-β [204]. Current research on radiation-induced pulmonary fibrosis has focused on EMT, Treg, cellular senescence and ROS production, with insufficient research on metabolic alterations, proteasomal and other pathways. Therefore, further investigation of the mechanisms of lung fibrosis in patients with thoracic tumors is needed.

### Radiation-induced skin fibrosis

Radiation-induced skin fibrosis is a common consequence of cancer radiotherapy and is caused by the prolonged presence of radiation dermatitis. Therefore, it is crucial to understand the mechanism of radiation-induced skin damage to enhance the effectiveness of cancer radiotherapy. In the study, some researchers examined the effect of ionizing radiation on gene expression profiles of human skin fibroblasts. This research indicate that genes associated with differentiation and inflammatory responses were active in irradiated human skin fibroblasts, where cell proliferation was inhibited [205]. Prolonged inflammation is an important mechanism for the development of radiation-induced fibrosis [3]. Inflammatory factors such as IL-1 and IL-6 have long been shown to promote the development of fibrosis [206].

According to research, TGF-β is a necessary pro-fibrotic signal for radiation-induced skin fibrosis to occur [207]. The interaction between stromal cell-derived factor-1α and its receptor CXCR4 activate the TGF-β/Smad signaling pathway through downstream MAPK signaling, ultimately leading to the promotion of fibrosis [208]. Inhibition of upstream platelet-derived growth factor receptor β has been found to reduce TGF-β expression, offering a promising anti-fibrotic pathway [209]. Another study has shown that low molecular weight fucoidan derived from brown seaweed can also inhibit TGF-β/Smad signaling [210]. Radiation-induced skin fibrosis is regulated by four core components, including EZR, MSN, CDC42 and ACTB, which play a significant role in the actin cytoskeleton [210]. A genome-wide analysis of radiation-induced skin fibrosis tissues revealed that ionizing radiation induces CpG dinucleotide demethylation in the exon 1 of the zinc transporter protein ZIP9, thereby recruiting the transcription factor Sp1 to promote ZIP9 expression [211]. Overexpression of ZIP9 activates the TGF-β/Smad signaling pathway and promotes the development of fibrosis, and Wnt/β-catenin is another classical pro-fibrotic pathway. Inhibition of this pathway has been found to attenuate radiation-induced skin fibrosis. Furthermore, Wnt/β-catenin also inhibits the conversion of epithelial and endothelial cells into myofibroblasts, which ultimately resists fibrosis [101].

Another group found that decreased methylation levels of diacylglycerol kinase α (DGKA) enhancer allow recruitment of the transcription factor early growth response 1 (EGR1), promoting increased radiation-induced DGKA expression [212]. The development of radiation-induced skin fibrosis is promoted by the epigenetic regulation of DGKA. To counteract this, de-epigenetic regulation of DGKA enhancers such as BET-bromodomain inhibitors are utilized to decrease the likelihood of radiation-induced skin fibrosis [213, 214].

### Radiation-induced liver fibrosis

During radiotherapy for hepatocellular carcinoma, the liver tissue being irradiated often undergoes fibrotic changes. HSCs are the primary instigators of liver fibrosis and they release a range of pro-fibrotic factors in response to radiation stimulation that encourage liver fibrotic changes [215]. TGF-β1 is one of them, which can promote HSC activation and liver fibrosis by activating downstream Smad signaling [216]. Like fibrosis in other organs, activated TGF-β1 signaling can further exacerbate radiation-induced liver fibrosis by stimulating ROS and CTGF production [110, 217]. The activation of the TGF-β signaling pathway requires the activation of Smad3 by TGF-β receptors. However, the process can be inhibited by high levels of intracellular Smad7 which in turn blocks the TGF-β/Smad pathway [218]. Therefore, targeted inhibition of TGF-β signaling can be a potential treatment for radiation-induced liver fibrosis [219]. In addition, a study showed that the Hedgehog signaling pathway may promote radiation-induced activation of hepatic stellate cells and play an important role in radiation-induced liver fibrosis [220].

The effects of radiation on cells can be manifested as DNA damage and oxidative damage. When exposed to ionizing radiation, mitochondrial DNA can be damaged, leading to ROS production, increased secretion of SASP and p53 activation, which eventually manifests as severe liver fibrosis [221]. Proteomics studies revealed that radiation can lead to the expression of α-SMA, CTGF, and other proteins. Additionally, bioinformatics analysis has revealed that radiation can increase the expression of the CDC20 gene [29]. When the CDC20 gene was knocked down, liver tissue showed an increase in p21 expression, which in turn inhibited HSC proliferation, HSC senescence and fibrosis [222, 223]. In epigenetic regulation, radiation promotes the expression of demethylase α-ketoglutarate-dependent dioxygenase ALKBH5 in hepatic stellate cells, resulting in toll-interleukin 1 receptor domain containing adaptor protein (TIRAP) mRNA demethylation, which in turn activates NF-κB signaling to promote hepatic stellate cell activation [224]. Increased expression of ALKBH5 resulted in the aggregation of monocytes and the promotion of macrophage M2 phenotype through the interaction of CCL5 and CCR5. This effect was further promoted by CCL5 secreted by M2 macrophages [224].

Radiation-induced liver fibrosis involves several non-coding RNAs that regulate its progression. Among them, circTUBD1 plays a crucial role as a regulator. In the presence of radiation, circTUBD1 forms a molecular sponge with miR-203a-3p, which in turn regulates the expression of Smad3. The increased Smad3 positively regulates circTUBD1, leading to the continuous accumulation of intracellular Smad3 and exacerbation of liver fibrosis [225, 226]. In addition, circTUBD1 also affects TLR4 signaling through miR-146a-5p, which promotes the secretion of pro-fibrotic factors such as IL-1β and TNF-α by post-illumination hepatic stellate cells [227]. For miR-146a-5p, it can inhibit *PTPRA* (protein tyrosine phosphatase receptor type A) gene expression, which leads to downstream SRC inactivation and inhibits radiation-induced fibrosis [228]. In all, radiation-induced liver fibrosis is a common toxic side effect in patients undergoing abdominal radiotherapy. The use of ionizing radiation for the treatment of liver cancer may also induce liver fibrosis is known to be a risk factor for liver cancer and should be minimized.

### Radiation-induced cardiac fibrosis

Radiation-induced cardiac fibrosis is a process in which large amounts of collagen are deposited in the heart after exposure to ionizing radiation. In addition to radiation-induced pulmonary fibrosis, radiation-induced cardiac fibrosis is another common toxic effect in patients treated with radiotherapy for thoracic tumors. Attempts to mitigate ionizing radiation-induced cardiac fibrotic changes will reduce the incidence of arrhythmias and heart failure. The conversion of cardiac fibroblasts into myofibroblasts is the main hallmark of radiation-induced cardiac fibrosis [229]. Like the previously described radiation-induced lung and skin fibrosis, TGF-β1 and CTGF are involved in the regulation of cardiac fibrosis [230]. In addition to activating TGF-β1/Smad3 signaling and promoting fibrosis progression, ionizing radiation may also regulate fibrosis through Smad-independent pathways such as PI3K/AkT and Rho/ROCK [231]. Peroxisome proliferator-activated receptor α (PPARα) is a member of the intranuclear receptor transcription factor superfamily that regulates the expression of target genes and has the function of regulating cardiac energy metabolism [232]. Transcriptomics and proteomics have shown that PPARα is involved in the regulation of radiation-induced cardiac fibrosis and is associated with TGF-β signaling [233]. PPARα is shown to exert an antifibrotic effect by inhibiting TAK1 phosphorylation in the Smad-independent TGF-β pathway. However, radiation has shown to decrease in PPARα expression, which in turn promotes TAK1 activation and downstream JNK and c-Jun activation, ultimately leading to the upregulation of fibrotic genes [234, 235]. The Smad-independent TGF-β pathway is the only one associated with PPAR’s ability to counteract radiation-induced fibrosis. However, the TGF-β/Smad pathway is not affected [234]. Growth differentiation factor 15, a member of the TGF-β superfamily is overexpression radiation-induced cardiac fibroblasts. This overexpression promotes radiation-induced cardiac fibrosis through the Smad-independent MAPK/ERK pathway [236].

Additionally, radiation-induced myocardial fibrosis is associated with cardiac energy metabolism. In a study comparing differentially expressed proteins before and after radiation-induced injury, it was discovered that the proteins primary associated with energy metabolism and fibrosis [156]. Subsequent research confirmed that radiation causes mitochondrial damage in cardiomyocytes, promoting the development of cardiac fibrosis. AMPKα is an intracellular energy receptor involved in the regulation of redox homeostasis [237]. Activation of AMPK has been shown to counteract the imbalance of oxidative stress in the heart caused by radiation, which can prevent myocardial fibrosis remodeling [238]. In a study using the agonist adropin, encoded by the energy homeostasis-related gene *Enho*, adropin promotes downstream PI3K/Akt signaling through activation of vascular endothelial growth factor receptor 2 [239]. Activated PI3K/Akt signaling decreases the expression of NOX4, eNOS and cleaved-Caspase 3, which attenuates radiation-induced myocardial fibrosis [239].

Oxidative stress is also an important factor in the regulation of fibrosis. Ionizing radiation causes an imbalance between oxidants such as NADPH oxidase and reductants such as superoxide dismutase, resulting in a large amount of ROS [240]. NADPH oxidases mainly include NADPH1-5, DUOX1 and DUOX2, are mainly responsible for this imbalance. Research has demonstrated that radiation can cause an upregulation of DUOX1/2 in cardiac tissue [241]. The induction of IL-4 or MAPK-mediated IL-13 can initiate this process [242, 243]. Furthermore, the expression of NOX2 and NOX4 is stimulated by radiation [241]. However, the excessive production of oxidase can lead to the overproduction of ROS, which can ultimately contribute to the development of myocardial fibrosis. In addition, the overproduction of ROS can also oxidize lipids, resulting in the production of lipid peroxides that can cause further damage to myocardial tissue and blood vessels [244, 245]. Vascular injury plays a crucial role in myocardial fibrosis, and abnormal platelet activation increases the risk of both vascular injury and radiation-induced myocardial fibrosis. However, the presence of platelet tissue factor pathway inhibitor-α can effectively inhibit the coagulation process, thereby reducing the severity of radiation-induced myocardial fibrosis [246]. The imbalance of oxidative stress affects immune cells and can indirectly regulate fibrosis. Ionizing radiation can induce a large amount of ROS, which activates the conversion of CD4^+^ T cells into Th2 lymphocytes. These lymphocytes secrete potent pro-fibrotic factors like IL-4 and IL-13 [247].

Non-coding RNAs are another important regulator of cardiac fibrosis. It was found that miR-21 is a pro-fibrotic factor that is highly expressed in response to radiation stimulation, and that high levels of miR-21 promote the expression of extracellular matrix regulatory genes GDF15, Grem1 and CCL7 [248]. A previous study identified a substance with antifibrotic effects, molecular hydrogen, which acts by decreasing miR-21 levels as well as increasing miR-15b levels [249]. Recent studies have highlighted the significance of sensory nerves in regulating radiation-induced cardiac fibrosis. These nerves can have a dual role in the process, acting as both anti-fibrotic agents by modulating mast cells and a pro-fibrotic role by increasing the expression of the injury-associated nuclear receptor NR4A1/2 [250]. Therefore, the effect of sensory nerves on radiation-induced fibrosis needs to be further investigated.

### Radiation-induced renal fibrosis

Radiation-induced renal fibrosis is a massive proliferation of fibrous tissue in the kidney caused by radiation, resulting in loss of renal function. It was previously reported that radiation can activate the renin–angiotensin–aldosterone system (RAAS), which in turn leads to vascular injury and promotes structural fibrosis in the kidney [251]. Radiation-induced oxidative stress imbalance is a significant mechanism of fibrosis. In addition, the excessive ROS oxidize lipids, which subsequently promote the expression of fibrotic factors [189]. These excess ROS cannot be scavenged, leading to DNA damage. The damaged DNA, in turn promotes apoptosis of kidney cells, further exacerbating radiation-induced renal fibrosis [252]. The fat-soluble vitamin coenzyme Q10 (CoQ10) may reduce excessive oxidative stress and lipid peroxidation, thus playing a role in reducing radiation-induced renal fibrosis [253]. Zinc is a micronutrient with antioxidant properties, and a recent study has found that zinc nanoparticles can be used to treat radiation-induced renal fibrosis by reducing oxidative stress [254]. Endoglin, a co-receptor for transforming growth factor is a potential pro-fibrotic factor. Mice with low expression of endoglin exhibit reduced levels of IL-1β and IL-6, both of which are pro-fibrotic factors [255].

### Radiation-induced intestinal fibrosis

The intestine is one of the most sensitive organs to ionizing radiation. Radiation-induced bowel injury often occurs during radiotherapy of patients with abdominal tumors. Like other organs, radiation-induced injury to the intestine is classified as acute and chronic. Acute radiation-induced injury manifests as an acute gastrointestinal syndrome such as intestinal mucosal rupture, bleeding, abdominal pain, and diarrhea, whereas chronic radiation-induced injury manifests as fibrosis [256]. Ionizing radiation damage to the intestinal mucosa is mainly caused by inducing inflammation of the small intestinal epithelium and apoptosis and cellular DNA damage [257].

Radiation-induced intestinal fibrosis is triggered by the interaction of multiple cytokines, immune cells, and enterocytes [258]. The pro-fibrotic factor CTGF adversely affects radiation-induced intestinal fibrosis, while attenuating Rho kinase signaling attenuates CTGF expression and inhibits fibrosis-like changes in intestinal smooth muscle cells [113, 259]. It is important to note that previous studies have not taken into consideration the role of intestinal flora in this relationship between intestinal fibrosis and ionizing radiation. While ionizing radiation causes intestinal fibrosis, it also leads to changes in the composition and β-diversity of the intestinal flora. Using nicotinamide mononucleotide (NMN) to treat mice with damaged intestines after irradiation, NMN was found to remodel the structure of the intestinal flora and alter its metabolic pathways, thereby reducing the extent of intestinal fibrosis [260].

Immune cells and their interactions with intestinal cells are also important pro-fibrotic factors. Fusion of bone marrow-derived macrophages with intestinal stromal cells promotes radiation-induced intestinal fibrosis [261]. Eosinophils also have been implicated in the regulation of radiation-induced intestinal fibrosis. Ionizing radiation causes intestinal crypt cell death, which leads to an increase in extracellular ATP levels, which in turn activates the secretion of C–C motif chemokine 11 (CCL11) by surrounding α-SMA-positive cells. CCL11 then interacts with CCR3 and recruits eosinophils [262]. In addition, activated α-SMA + cells promote eosinophil expression of TGF-β1 through granulocyte–macrophage colony-stimulating factor signaling and promote the development of radioactive intestinal fibrosis [262]. Lgr5^+^ intestinal stem cells have been identified as playing a crucial role in regenerative repair of radiation-induced intestinal injury [263], but their involvement in fibrosis remains unclear.

## Diagnosis of radiation-induced fibrosis

The progression and severity of fibrotic disease is contingent upon the amount of fibrosis present within the organ [264]. As such, early detection and diagnosis of fibrosis is crucial. The gold standard for diagnosing fibrosis is through histology, which is typically obtained through fine-needle biopsies or surgical specimens [265]. Histologic scoring methods vary from organ to organ and usually depend on the etiology of the fibrosis. However, histologic assessment of fibrosis still has serious limitations, such as percutaneous liver or kidney biopsy, which carries risks including bleeding [266]. Biopsy results may represent only a small fraction of the organs in which fibrosis is distributed [267]. As a result, many non-invasive methods have been developed. Some of them are used in clinical practice to assess the progressive stages of fibrosis. These techniques include imaging methods, mechanical and functional tests, serum biomarkers, and genetic risk factors for fibrosis.

### MRI and CT

Imaging techniques are widely used in patients with chronic diseases where fibrosis is suspected. In the liver, MRI-derived proton density fat fraction has been widely used to quantify liver fat content and has been the focus of early clinical trials of antifibrotic drugs in non-alcoholic steatohepatitis [268]. Cardiac MRI is an essential tool in clinical algorithms due to its ability to assess various aspect of the heart such as cardiac wall thickness, myocardial mass, late gadolinium enhancement and myocardial extracellular volume fraction. The latter is particularly important as it is closely linked to myocardial fibrosis, making cardiac MRI a valuable diagnostic tool for detecting and monitoring heart conditions [269]. Regarding pulmonary fibrosis, high-resolution chest computed tomography (CT) provides diagnostic and prognostic information in patients with pulmonary fibrosis, and various radiologic parameters have been established to stage disease severity, making lung biopsy rarely necessary [270]. For example, homogeneous ground-glass attenuation, representing early radiation pneumonitis, can be detected on CT imaging a few weeks after completion of therapy, even in the absence of findings on chest radiography [271–273].

### Elastography techniques

New elastography techniques are available for the assessment of liver fibrosis. These methods include ultrasound-based transient elastography (TE, also called fibroscan), acoustic radiation force impulse imaging (ARFI) and shear wave elastography (SWE), and MRI-based elastography (MRE) [265]. These methods have been widely used in patients with chronic liver disease, and this technique has been recommended for use in the diagnosis of liver fibrosis [274]. For renal fibrosis, similar ultrasound and MR elastography approaches are currently being adapted [264]. In contrast, cardiac fibrosis can be typically diagnosed by functional testing and traditional methods such as volumetric tracings, echocardiography or cardiac MRI.

### Biomarkers

Although circulating biomarkers of fibrosis are simple, reproducible and inexpensive compared to histological, imaging or mechanical assessments, their major drawbacks include a general lack of specificity for fibrosis, a large overlap between stages of fibrosis progression, and the presence of many confounding factors of individual markers. In pulmonary fibrosis, fibrosis-related factors such as chemokines (IL-8, CCL18), proteases (MMP-1, MMP-7) or growth factors have been suggested to have diagnostic potential for fibrosis, but their utility in clinical routine has not been established [275]. In renal fibrosis, potential renal fibrosis biomarkers include TGF-β, monocyte chemoattractant protein-1 (MCP-1) and MMP-2, or urinary excretion of collagen III and related proteins such as the N-terminal pro-peptide of type III collagen [276]. In the specific field of liver fibrosis, numerous biomarkers have been suggested and are currently being utilized to a certain degree. These include hyaluronic acid and the N-terminal pro-peptide of type III collagen, also known as PIIINP [265]. The best diagnostic and prognostic accuracy can be achieved when biomarkers are used in predictive models and when different risk factors are integrated with information from multiple biomarkers [277].

In the realm of fibrosis biomarker research, there are several innovative to explore, including mitochondrial DNA, circulating miRNA, lncRNA and microbiome signatures found in stool samples [275]. A promising avenue of investigation involves identifying biomarkers that accurately reflect the pathogenic process of fibrogenesis or fibrolysis. The different collagens and collagen fragments that are being released into the circulating might therefore have exceptional potential as biomarkers [278]. New imaging approaches that combine visualization with molecular targeting have already shown promising results in molecular MRI for animal models of liver, kidney, heart and lung fibrosis [279].

### Diagnosis by PET-CT

The use of radionuclides to detect fibrotic lesions is an emerging field. Positron emission tomography (PET)-CT is a relatively advanced imaging technique in the field of nuclear medicine. It can reflect the signal of lesions by detecting the concentration of radiotracer in the lesion area [280]. ^18^F-fluciclatide is a radiotracer that can bind to α_v_β_3_ integrin transmembrane receptors and activate extracellular matrix modifications. Its uptake by tissues can reflect the activity of fibrosis, but also angiogenesis and inflammatory responses, which greatly reduces the specificity of ^18^F-fluciclatide [281]. In recent years, studies have focused on fibroblast activating protein. This is a protein that is low expressed in normal tissues and significantly increased in fibrotic tissues and is an ideal target to mark and detect myofibroblast activation [282, 283]. Based on this, radiolabeled fibroblast activation protein inhibitor was used to assess and quantify the expression of fibroblast activating protein [284]. The [^68^ Ga] Ga-FAPI-04 PET-CT was originally used for tumour imaging, but an increasing number of studies are now demonstrating its potential value in the diagnosis of cardiac, pulmonary, renal, intestinal and hepatic fibrosis, with the advantage of being non-invasive and sensitive [14, 285–288]. In addition, fibrosis occurs later than fibroblast activation and FAP expression; therefore, the use of [^68^ Ga] Ga-FAPI-04 PET-CT facilitates the early diagnosis of fibrosis [286, 289]. Interestingly, a recent study used PET-MR in combination with [^68^ Ga] Ga-FAPI to detect intestinal fibrosis in patients with Crohn's disease [290]. Currently, radiotracers have not been used to detect radiation-induced fibrosis. And the technique is only in preclinical studies and more evidence is needed to prove its clinical application.

## Advances in the treatment of radiation-induced fibrosis

Increasingly advanced modern radiotherapy has virtually minimized exposure to radiation of normal tissues, but radiation-induced fibrosis is still a common and severe adverse chronic event. Although amifostine is so far the only thiol derivative approved by Food and Drug Administration (FDA) for treatment of radiation-induced damage, its use is limited for wide clinical application due to toxicity at the therapeutic dose. Unfortunately, at present, no more effective measures can completely alleviate or reverse tissue fibrosis, so symptomatic treatments for fibrosis-related symptoms are commonly adopted, like non-steroidal anti-inflammatory drugs (NSAIDs) for muscular pain, injection of botulinum toxin for dystonia, and oxygen therapy or mechanical ventilation for respiratory failure. Therefore, prevention is the crucial first step in mitigating radiation-induced fibrosis. In addition to careful consideration of the dose and timing of radiotherapy, it is important to eliminate any identified risk factors. Smoking, for example, has been linked to a significantly increased incidence and severity of radiation-induced fibrosis, likely due to impaired oxygenation and elevated carboxyhemoglobin levels [291, 292]. For decades, a variety of medications have been studied for the treatment or prevention of radiation-induced tissue fibrosis and most of them yielded promising results (Table 1).

**Table 1:** Current medications studied for radiation-induced fibrosis

Name	Molecular type	Target/action	Site	Stage of project trial	References
SOD-TAT	Ligand-modified SOD	Scavenging ROS and free radicals	Lung	Preclinical study	[296]
AEOL10113	Nonenzymatic SOD mimic	Suppressing redox-regulated pathways	Lung, rectum, prostate	Preclinical study	[300, 302, 305]
Melatonin	Indole-derived hormone	Scavenging ROS and RNS and activating antioxidative enzymes	Lung	Preclinical study	[307]
Combined PTX and Vit E	A methylxanthine derivative and a vitamin	antioxidant, anti-inflammatory and suppressing the TGF-β pathway	Neck and chest	In clinical use	[314–317]
Triptolide	Diterpenoid epoxide	Inhibiting the activity of alveolar macrophages and the IKKβ/NF-κB pathway	Lung	Preclinical study	[324]
SKI2162, EW-7197, LY2157299, LY2109761	TGFβR I inhibitor	Blocking TGF-β receptor I	Lung, skin	Preclinical study	[325–328]
P144	Hydrophobic peptide	Binding to soluble TGF-β1	Muscle	Preclinical study	[329]
Imatinib	Receptor tyrosine kinase inhibitor	Blocking PDGF receptor	Lung, skin	Preclinical study	[331]
Pamrevlumab	Monoclonal antibody	Blocking CTGF receptor	Lung	Preclinical study	[105]
sLRP6E1E2	Soluble Wnt receptor	Binding to extracellular Wnt ligands	Skin	Preclinical study	[101]
Curcumin	Natural polyphenol	Activating antioxidative enzymes, inhibiting NF-κB pathway and downregulating inflammatory factors	Lung, skin	Preclinical study	[364, 366]
Rosmarinic acid	Natural polyphenol	Inhibiting RhoA/Rock pathway	Lung, parotid glands	Preclinical study	[370, 371]
GSPs	Plant extract	Ameliorating mitochondrial dysfunction and inhibiting MAPK pathway	Lung, breast	In clinical trials (NCT00041223)	[377]
Pirfenidone	Pyridone analog	Inhibiting TGF-β1/Smad3 pathway	Lung, skin	In clinical trials (NCT03902509)	[385]
Nintedanib	Receptor tyrosine kinase inhibitor	VEGF receptor, FGF receptor and PDGF receptor	Lung	Preclinical study	[386]
Esomeprazole	Proton pump inhibitor	inducing of MAPK/Nrf2/HO1 pathway and inhibiting of DDAH/iNOS pathway	Skin	Preclinical study	[345]
Metformin		activating AMPK and Inhibiting respiratory chain complex I	Lung, heart, skin	Preclinical study	[390–394]
Losartan	ACEI	inhibiting TGF-β/Smad signaling pathway	Heart, breast	In clinical trials (NCT05637216) (NCT05607017)	[354]
Deferoxamine	Iron chelator	Inhibiting HIF-1α	Skin	Preclinical study	[356–358]
Methoxyestradiol	Metabolite of estradiol	inhibiting HIF-1α	Lung, skin	Preclinical study	[359, 361]
MSCs		secreting anti-inflammatory and antifibrotic factors	Lung, parotid glands	In clinical trials (NCT02513238) (NCT02277145)	[400]

*SOD* superoxide dismutase; *PTX* pentoxifylline; *Vit E* vitamin E; *GSPs* Grape seed proanthocyanidins; *MSCs* Mesenchymal stem cells

### Antioxidant treatment

Oxidative stress is one of the central pathophysiological mechanisms in radiation damage, so lots of radio-prophylactics are developed based on free radical scavenging or antioxidation. SOD is the most principal endogenous defense against oxygen radicals [293]. The clinical application of SOD is limited due to its large molecular weight, which prevents it from passing through the cell membrane freely [294]. Additionally, a recent study found that the topical SOD was not effective in treating post-radiation fibrosis [295].

Over recent years, varied ligand-modified SODs and nonenzymatic SOD mimics have been synthesized to overcome the handicaps of native SODs. HIV-1 TAT protein transduction domain (YGRKKRRQRRR) is usually utilized to carry larger molecules across the cell membrane. A recombinant protein SOD-TAT that fuses hCuZn-SOD with TAT enhanced pulmonary antioxidant ability and thus reduced radiation-induced pulmonary fibrosis [296]. Furthermore, a bifunctional antioxidant enzyme named GST-TAT-SOD fused with glutathione-S-transferase (GST) and TAT-SOD was constructed, which manifested a strong radioprotective activity against whole-body irradiation in mice [297].

A series of Mn (III) porphyrin-based SOD mimics such as MnTE-2-PyP^5+^ (AEOL10113) and MnTDE-2-ImP^5+^ (AEOL10150) have been assessed in various preclinical models as radioprotectants. Several studies suggested that these Mn porphyrins ameliorated radiation-induced lung and rectum fibrosis by suppressing redox-regulated pathways including HIF-1α, NF-κB, and TGF-β [298–302]. Previous studies have suggested that Mn porphyrins protect normal tissues from radiation-induced damage, and act as chemo-/radio-sensitizers in tumor cells [303, 304]. However, a study showed that AEOL-10150 increases the survival in lethal lung radiation injury in rhesus macaques but does not improve the incidence of pneumonitis or the severity of fibrosis [305].

Melatonin has been identified as a potent antioxidant that can directly scavenge ROS and RNS. Additionally, it can indirectly stimulate antioxidative enzymes and suppress pro-oxidative enzymes [306]. Numerous studies have demonstrated that melatonin can reduce radiation-induced pneumonitis and lung fibrosis [307, 308]. In addition, the ease with which melatonin penetrates all cell types and low toxicity attributed to the instinctive metabolic pathway might be the natural advantage of melatonin. Meanwhile, in vitro studies have revealed the antitumor activity of melatonin when used alongside irradiation [306].

Other kinds of antioxidant nutrients such as α-lipoic acid [309, 310] and coenzyme Q10 [311] are also demonstrated to be beneficial to radiation-induced fibrosis in preclinical models. Although the protective effect of antioxidants such as α-tocopherol (vitamin E; Vit E) during radiation therapy is well-established in radiation-induced mucositis, their effects on the human body are not yet fully understood. One essential concern is to safeguard normal tissues from radiation damage while not interfering with tumor treatment. However, previous studies have highlighted that the use of high doses of antioxidants like vitamin E; Vit E during radiation therapy may give rise to a higher local recurrence rate in patients with head and neck tumors [312, 313].

### Anti-inflammatory treatment

Plentiful studies demonstrated that combined treatment with anti-inflammatory pentoxifylline (PTX) and vitamin (Vit) E can reverse radiation-induced cervicothoracic fibrosis by suppressing the TGF-β pathway. Marked efficacy can be observed clinically after three months of treatment with combined PTX (800 mg/d) and Vit E (1,000 IU/d). Fibrosis continued to be alleviated along with the time of treatment, and the mean time to maximal treatment effect was 24 months [314, 315]. There was a risk of a rebound effect if treatment was too short. Thus, treatment of fewer than 3 months is not recommended in patients with severe radiation-induced fibrosis [316]. A study, in which the median time from radiotherapy to the trial showed that 6-month treatment of Vit E and pentoxifylline did not provide any benefits [317]. Poor efficacy is presumably ascribed to relatively shorter treatment and more severe or older established fibrosis. Additionally, combined treatment with PTX and Vit E started 1–3 months after radiotherapy has been proven to prevent fibrosis [318]. Recently, Erbium: YAG fractional laser ablation has been exploited for cutaneous co-delivery of PTX and d-α-tocopherol succinate. This method has shown to temporarily impair the stratum corneum and increase the bioavailability of PTX in the skin potentially providing an effective alternative to systemic exposure [319, 320]. Triptolide (TPL) is an herb-purified diterpenoid epoxide with potent anti-inflammatory and immunosuppressive effects. TPL mitigated radiation-induced pulmonary fibrosis in a mouse model, and possible mechanisms have been explored [321, 322]. TPL could inhibit the infiltration, Inflammatory factors secretion, and phagocytosis of alveolar macrophages and eventually decrease paracrine activation of myofibroblasts [323]. TPL also inhibited the IKKβ/NF-κB pathway and thus reduces LOX production in fibroblasts [324].

### Targeted treatment for fibrosis

#### Targeted treatment for the TGF-β1 signaling pathway

As we discussed earlier, the TGF-β1 signaling pathway plays a crucial role in the development of fibrosis. Targeted inhibition of these correlated signaling pathways is a promising therapeutic method. Several novel TGF-β1 receptor kinase inhibitors, including SKI2162 [325], vactosertib (EW-7197) [326], galunisertib (LY2157299) [327] and LY2109761 [328], are demonstrated to specifically block the TGF-β1 signaling pathway and hence suppress radiation-induced murine pulmonary and skin fibrosis. P144 is a hydrophobic peptide that can directly binds to soluble TGF-β1, thus block the binding of TGF-β1 to the TGF-β1 type I receptor. Intravenous administration of P144 clearly reduced radiation-induced muscle fibrosis in rabbits [329]. Some prospective studies of topical P144 in patients with systemic sclerosis have been completed, but these inhibitors of TGF-β signaling pathway have not been tested for efficacy on radiation-induced fibrosis on humans.

#### Targeted treatment against the PDGF signaling pathway

In a study, several receptor tyrosine kinase inhibitors (RTKIs) that target platelet-derived growth factor receptor-α (PDGFR-α) and PDGFR-β were also found to reduce lung fibrosis after radiation injury and prolong animal survival [330]. Among these inhibitors, Imatinib, commonly used for the treatment of chronic myelogenous leukemia and gastrointestinal stromal tumors, was shown to attenuate radiation-induced pulmonary fibrosis in mice whether it was administered before radiation or after acute inflammation [331]. In irradiated skin, treatment with imatinib was found to not only reduce the activation of PDGFR but also decreased the levels of TGF-β [209]. Combing the inhibition of TGF-β1 and PDGF signaling may be of more positive effect [327].

#### Targeted treatment against CTGF

CTGF plays a critical role in radiation-induced fibrogenesis. Pamrevlumab (FG-3019) is a fully human recombinant monoclonal antibody against CTGF and is in an ongoing phase 3 trial (NCT03955146) in patients with idiopathic pulmonary fibrosis (IPF) [332]. Blocking CTGF with FG-3019 could prevent and reverse pulmonary fibrosis after thoracic irradiation with prolonged survival and improved lung function in mice. A significant finding is that lung remodeling, which had already occurred by 16 weeks after irradiation, was still reversed and lung function was restored with FG-3019 treatment [115].

#### Targeted treatment against the Rho/ROCK signaling pathway

Targeting of Rho/ROCK signaling pathway is another way to inhibit the effect of CTGF. Many preclinical studies have shown that ROCK inhibitors alleviate pulmonary fibrosis through a variety of potential mechanisms [201]. Statins are discovered to downregulate CTGF expression by inhibition of Rho protein is prenylation, and thereby repress fibrosis [333]. In preclinical models, both pravastatin and lovastatin have been shown to reduce radiation-induced fibrosis [334–337]. A prospective trial was conducted to evaluate the effectiveness of pravastatin treating with delayed cutaneous and subcutaneous grade-2 radiation-induced fibrosis in patients. In a study involving 60 patients, pravastatin 40 mg/d for 12 months for 12 months. The results demonstrated that this treatment successfully reversed established radiation-induced cutaneous and subcutaneous fibrosis, as evidenced by a decrease in thickness and clinical signs of fibrosis [338].

#### Targeted treatment for the Wnt/β-catenin signaling pathway

The Wnt/β-catenin signaling pathway is a significant target in fibrotic reactions and there have been numerous strategies to specifically block this pathway. XAV939 can preserve the β-catenin degradation complex’s integrity, which ultimately result in the degradation of β-catenin and the inhibition of Wnt/β-catenin signaling. It was shown that XAV939 significantly improved the pulmonary fibrosis induced by bleomycin and increased mouse survival [339]. In another study, researchers found that radiation-induced dermal fibrosis can be improved by inhibiting the Wnt/β-catenin signaling pathway. This was achieved by adenovirus gene therapy, which expressed a Wnt antagonist that binds to Wnt ligands [101].

#### Proton pump inhibitors

Clinical data revealed that proton pump inhibitors (PPIs) are associated with favorable outcomes in patients with IPF and, in fact, antioxidant and anti-inflammatory activity of PPIs has been reported in preclinical models [340, 341]. Previous research demonstrated the pleiotropic effect of esomeprazole, which significantly suppressed lung inflammation and fibrosis in vivo and in vitro through induction of the MAPK/Nrf2/HO1 pathway and inhibition of the DDAH/iNOS pathway [342–344]. In addition to assessing the effectiveness of an esomeprazole topical product, also known as dermaprazole on radiation-induced dermatitis, the study found that dermaprazole was able to speed up the healing process of wounds and reduce scarring on the irradiated skin [345]. Another recent study revealed that esomeprazole can enhance the anti-fibrotic efficacy of pirfenidone through complementary molecular mechanisms [346].

#### Angiotensin-converting enzyme inhibitors

Angiotensin-converting enzyme inhibitors (ACEIs) have been shown to impact fibrosis in various tissues, although the exact mechanism remains unclear. It may be linked to the inhibition of the TGF-β1/Smad signaling pathway [347]. Clinical studies have confirmed the effectiveness of losartan in treating of IPF and keloid [348, 349]. Some kinds of ACEIs mitigated fibrosis after thorax irradiation in preclinical models [350–353]. A retrospective study showed that ACEIs decrease the risk of radiation pneumonitis in lung cancer patients receiving thoracic irradiation [354]. There have been two undergoing clinical trials that aim to investigate whether losartan can prevent breast (NCT05637216), or cardiac muscle (NCT05607017) fibrosis induced by radiotherapy in patients with breast cancer.

#### HIF-1α inhibitors

Deferoxamine (DFO), an iron chelator approved for treating iron-overload-associated diseases, can limit the iron-dependent generation of ROS and the degradation of HIF-1α. HIF-1α is a transcription factor that upregulates pro-angiogenic genes, including vascular endothelial growth factor (VEGF) and endothelial nitric oxide synthase (eNOS). It has been shown that DFO mitigates radiation-induced hypovascularity and improves bone regeneration and soft tissue elasticity [355, 356]. Topical DFO has been developed for skin application and has been shown to improve cutaneous vascularity and tissue pliability in an irradiated nude mouse model [357]. In comparison to treatment after recovery from irradiation, prophylactic treatment with transdermal DFO has been found to be more effective in decreasing levels of markers of ROS and apoptosis, improving tissue perfusion, and mitigating radiation-induced skin fibrosis [358]. Another HIF-1α inhibitor, 2-Methoxyestradiol (2-ME), which is a metabolite of estradiol, has been approved as an anti-cancer drug that inhibits tubulin polymerization [359]. In a study conducted on irradiated mice, it was found that 2-ME had positive effects on radiation-induced pulmonary injury and fibrosis. The mice model improved survival rates and better pulmonary functions. These effects were attributed to the inhibition of radiation-induced inflammation and vascular EndMT which depends on HIF-1α [30, 360, 361]. The protective effects of 2-ME were also observed in the skin of irradiated mice on the vascular endothelium.

### Natural products

In recent years, there has been a growing interest in natural products that have healthcare benefits. These products are often considered to be more cost-effective, less toxic, and have a wider therapeutic window than l artificial drugs. Several plant extracts that contain antioxidant polyphenols have been found to have both radio-sensitization and radioprotection effects, making them a safe and effective adjuvant in radiotherapy. Curcumin (diferuloylmethane) ameliorated radiation-induced pulmonary, heart, liver, and skin damage and fibrosis, but does not protect tumor cells from radiation killing [362–366]. At present, randomized controlled trials have been conducted to evaluate the efficacy of curcumin to treat radiation dermatitis and oral mucositis. Curcumin mouthwash was equally effective and safe in prevention of the radiation-induced oral mucositis as the standard medication benzydamine (a kind of NSAID) [367]. Oral curcumin, compared with placebo, did not demonstrate a detectable benefit for radiation dermatitis in breast cancer patients [368, 369]. Rosmarinic acid (RA) substantially reduced radiation-induced fibrosis of the parotid glands and lungs. The possible mechanism is that RA upregulates miR-19b-3p, which targets myosin phosphatase target subunit 1 (MYPT1), and thereby attenuates RhoA/Rock signaling [370, 371]. Grape seed proanthocyanidins (GSPs) have been found to protect against radiation damage to various tissues by ameliorating mitochondrial dysfunction and suppressing MAPK Signaling Pathway [372–376]. However, a phase 2 trial administering GSPs orally failed to show efficacy in treating breast induration in patients who underwent radiotherapy for breast cancer [377]. In addition, there are also quercetin [378] and its monoglycoside [379], naringenin [380], and resveratrol [381], all of which are shown with radioprotective and antifibrosis effects. The exact pharmacological mechanisms are still unclear though, the extensive biological functions of these natural products may just match the intricate mechanisms of fibrosis.

### Pirfenidone and nintedanib

The pathophysiologic events induced by radiation are like those that occur in idiopathic pulmonary fibrosis (IPF) [328]. Both pirfenidone and nintedanib are small-molecule oral drugs and their ability of retarding the rate of progression of IPF have been approved by FDA. Unfortunately, these two drugs do not completely stop the progression of IPF [382]. Pirfenidone selectively downregulates the expression of TGF-β1, PDGF, and other fibrosis-associated cytokines, and nintedanib is a tyrosine kinase inhibitor targeting the VEGF receptor, FGF receptor, and PDGF receptor. Animal experiments have shown that pirfenidone alleviates radiation-induced pulmonary and intestinal fibrosis and reduces the expression of the TGF-β1/Smad3 signaling pathway [107, 383, 384]. According to a small-scale pilot study treatment with Pirfenidone resulted in a slight improvement in limited activity caused by after-radiotherapy fibrosis [385]. A phase 2 clinical trial (NCT03902509) is now underway and aims to confirm the efficacy of pirfenidone in the treatment of post-radiation lung injury. Nintedanib histologically reduced radiation-induced lung fibrosis in mice [386]. There have been two phase 2 clinical studies that aim to evaluate the effect of nintedanib on radiation pneumonia. One of the two (NCT02452463) was terminated prematurely owing to low accrual, while another (NCT02496585) is still ongoing. However, nintedanib inhibits wound healing. A concerning case study reported, a patient with head and neck cancer who was undergoing radiotherapy and was also taking nintedanib for their IPF. This patient experienced a significant delay in their recovery from radiation mucositis, highlighting the potential negative effects of nintedanib on wound healing [387]. This might suggest that nintedanib should not be used during the acute injury and repair phase after radiotherapy.

### Metformin

Metformin is the most widely prescribed medicine to treat type 2 diabetes mellitus, also manifests radio-sensitization for tumors and radioprotection for normal tissue [388]. Targeting mitochondria and subsequent activation of AMPK are considered the hinge of pleiotropic actions of metformin [389]. Recent studies indicate that metformin can attenuate radiation-induced fibrosis in the lung [390–392], heart [393] and skin [394]. The proposed explanation suggests that metformin candecrease mitochondrial ROS production by inhibiting of respiratory chain complex I, activate the DNA repair pathway through AMPK activation, and reverse the upregulation of NADPH oxidases such as NOX4 and DUOX1/2 induced by radiation. While there have been several randomized clinical trials aimed at testing the radio-sensitization effect of metformin, there has yet to be a clinical study specifically designed to test its radioprotective effects.

### Mesenchymal stem cells

Mesenchymal stem cells (MSCs) are multipotent stem cells that can be isolated from numerous stromal tissues within the body and can differentiate into adipocytes, chondroblasts or osteoblasts in vitro. MSCs exhibit immunomodulatory and paracrine functions along with low immunogenicity and easy accessibility. Adipose-derived MSCs (Ad-MSCs), bone marrow-derived MSCs (BM-MSCs), and umbilical cord-derived MSC (UC-MSCs) are the commonly used sources for the extraction of MSCs. In recent years, there have been lots of preclinical studies demonstrating that the production of MSCs plays a crucial role in radiation-induced fibrosis. In a study conducted on mice, it was found that systemic infusion of BD-MSCs altered the phenotype of macrophages and suppressed local inflammation in a TNF-receptor 2-dependent manner. This resulted in the inhibition of cutaneous radiation-induced fibrosis [125]. Similarly, Ad-MSCs were found to attenuate radiation-induced murine colon fibrosis by releasing hepatocyte growth factor (HGF) and tumor necrosis factor-stimulated gene 6 (TSG-6) [395]. The anti-fibrotic effects of Ad-MSCs on irradiated lungs by stimulating the endogenous secretion of HGF and PGE2 [396]. It was recently found that Ad-MSCs medium inhibited the induction of EMT via Wnt/β-catenin signaling in vitro and in vivo, which was mediated by Ad-MSCs-secreted DKK-1, a potent antagonist of the canonical Wnt pathway [106]. A study illustrated that injection of Ad-MSCs within two hours and one week of thoracic irradiation resulted in beneficial outcomes in the treatment of radiation-induced pulmonary fibrosis compared to one-time injection [397]. In addition, UC-MSCs modified with SOD3 or decorin (DCN) are found to show more beneficial effects on radiation-induced fibrosis compared to conventional MSCs. These cell-based gene therapies can realize the combined delivery of UC-MSCs and SOD3 or DCN, which are respectively an antioxidant enzyme in extracellular spaces and a natural inhibitor of TGF-β signaling. Meanwhile, administration of these UC-MSCs within hours after irradiation resulted in much better improvement than that after dozens of days [398, 399]. MSCs are tested clinically for the treatment of radiation-induced xerostomia (NCT02513238) and pulmonary fibrosis (NCT02277145). However, in addition to these promising results, we must confront the current limitations of MSC-based therapy. Safety is the most concerned issue as the pro-tumorigenic effect is found in MSCs [400]. Fortunately, from the existing clinical data, MSC-based therapy may be safe. The heterogeneity of MSCs that impacts quality control is another of the major obstacles to the realization of clinical application at present. Besides, there is no sufficient data that fully answer problems such as the optimal source and culture conditions of MSCs, and the best route and dose of administration. In short, the feasibility and efficacy of MSCs in the treatment of radiation-induced fibrosis in humans are difficult to be confirmed in the short term.

## Conclusions and future directions

Although radiotherapy technology has advanced to a point where accuracy can be precisely controlled, the surrounding normal tissues are still affected by radiation. The current understanding of fibrosis mechanisms has primarily centered around oxidative stress, cytokines, and cellular interactions related to fibrosis. In addition, the emerging roles of metabolic reprogramming, the cGAS/STING pathway, and the proteasome pathway in radiation-induced fibrosis have been extensively studied. Radiation-induced fibrosis in each organ has many common mechanisms of occurrence as well as its own unique mechanisms. Myofibroblasts are the main body of fibrosis. Factors that activate myofibroblasts can essentially cause extracellular matrix remodeling and fibrosis to occur. For example, TGF-β, IL-1β, CTGF, and other fibrosis-promoting factors are present in various organs, including the heart, liver, lung, kidney, skin, and intestine. It is noteworthy that some mechanisms regulating fibrosis are bidirectional. For example, SASP secreted by senescent macrophages can promote the development of fibrosis, but senescence of myofibroblasts leads to the loss of the myofibroblast phenotype. In this paper, in addition to the mechanism of ionizing radiation-induced fibrosis, we also explain the mechanism of fibrosis induction by other factors such as bleomycin and unilateral ureteral ligation. We found that some cells and some signaling pathways involved in radiation-induced fibrosis and non-radiation-induced fibrosis may be the same. For example, they both need to promote fibrosis formation by activating fibroblasts. In addition, they can both promote the development of fibrosis through signaling pathways such as TGF-β and Wnt/β-catenin. However, the commonality of the two still needs to be proved by much research. Research on non-radiation-induced fibrosis is relatively more abundant. This may provide some research ideas for radiation-induced fibrosis.

Radiation-induced fibrosis is a late toxic side effect of radiotherapy received by cancer patients. Given the adverse health effects of fibrosis, early diagnosis is crucial. In recent years, in addition to traditional imaging modalities such as serum marker testing, histological examination, CT and MRI, nuclear medicine techniques have opened a new era to achieve fibrosis visualization. For example, the effective combination of PET and radiotracer has enabled non-invasive, efficient and sensitive fibrosis diagnosis, making early fibrosis easier to detect. Unfortunately, traditional histologic and serologic tests lack specificity and sensitivity. Also, the emerging nuclear medicine technology is currently only in the preclinical research stage. Finally, the lack of understanding of the mechanisms involved has hindered the development of effective therapeutic tools. Presently, available treatments primarily rely on anti-inflammatory and antioxidant treatments, but their efficiency is very limited, and it is difficult to reverse the organ fibrosis and organ dysfunction that has occurred. Some targeted therapies and stem cell therapies, such as mesenchymal stem cells, show great promise for clinical application. However, much work remains to be done to understand the mechanisms underlying the development of radiation-induced fibrosis to develop precise and effective therapies.

## Abbreviations

RIF: Radiation-induced fibrosis
ROS: Reactive oxygen species
ECM: Extracellular matrix components
RIHD: Radiation-induced heart disease
TGFs: Transforming growth factors
ILs: Interleukins
α-SMA: α-Smooth muscle actin
MMPs: Matrix metalloproteinases
TIMPs: Tissue inhibitors of metalloproteinases
HSC: Hepatic stellate cell
EMT: Epithelial-mesenchymal transition
EndoMT: Endothelial-mesenchymal transition
CSF1R: Colony-stimulating factor receptor-1
CSF1: Colony-stimulating factor 1
CTGF/CCN2: Connective tissue growth factor
PDGF: Platelet-derived growth factor
MMT: Macrophage-myofibroblast transition
Treg: Regulatory T cell
SASP: Senescence-associated secretory phenotypes
FBW7: F-box and WD40 repeat domain-containing 7
TPP1: Telomeric protection protein 1
LXA4: Lipoxin A4
HIF-1α: Hypoxia-inducible factor-1α
MAPK: Mitogen-activated protein kinase
AMPK: AMP-activated protein kinase
NOX4: NADPH oxidase 4
CUGBP1: CUG-binding protein 1
TBK1: TANK-binding kinase 1
LRP5/6: Low-density lipoprotein receptor-associated protein 5/6
WISP1: Wnt-1-induced secreted protein
RNS: Reactive nitrogen species
NLRP3: NOD-like receptor protein 3
Nrf2: Nuclear factor erythroid-2-related factor 2
Keap1: Kelch-like ECH-related protein 1
RIPK3: Receptor-interacting protein kinase-3
SOD: Superoxide dismutase
STING: Stimulator of Interferon Genes
cGAS: Cyclic guanosine adenosine phosphate synthase
cGAMP: Cyclic dinucleotide 2′3′ cyclic GMP-AMP
IRF3: Interferon regulatory factor 3
XBP1: X-box binding protein 1
BINP3: BCL2/adenovirus E1B interaction protein 3
DNMT: DNA methyltransferase
PDK1: Pyruvate dehydrogenase 1
GLUT1: Glucose transporter protein 1
mTORC1: Rapamycin complex 1
FAO: Fatty acid oxidation
STAT6: Signal transducer and activator of transcription 6
UPS: Ubiquitin–proteasome system
DUB: Ubiquitin-dissociating enzyme
YAP: Yes-associated protein
FAT10: F-associated transcript 10
FPS: FAT10-proteasome system
ALKBH5: Alkylation repair homolog 5
FOXM1: Forkhead box M1
MZF1: Myeloid zinc finger protein 1
BCL6: B-cell lymphoma 6 protein
SIRP3: Sphingosine-1-phosphate receptor 3
ROCK: Rho-associated convoluted helix kinase
DGKA: Diacylglycerol kinase α
PPARα: Peroxisome proliferator-activated receptor α
RAAS: Renin–angiotensin–aldosterone system
CoQ10: Coenzyme Q10
NMN: Nicotinamide mononucleotide
CCL11: C–C motif chemokine 11
CAF: Cancer-associated fibroblast
FSTL1: Follistatin-like 1
PET: Positron emission tomography
Vit E: Vitamin E
PTX: Pentoxifylline
TPL: Triptolide
RTKIs: Receptor tyrosine kinase inhibitors
IPF: Idiopathic pulmonary fibrosis
RA: Rosmarinic acid
GSPs: Grape seed proanthocyanidins
PPIs: Proton pump inhibitors
ACEIs: Angiotensin-converting enzyme inhibitors
DFO: Deferoxamine
VEGF: Vascular endothelial growth factor
eNOS: Endothelial nitric oxide synthase
2-ME: Methoxyestradiol
MSCs: Mesenchymal stem cells
Ad-MSCs: Adipose-derived mesenchymal stem cells
BM-MSCs: Bone marrow-derived mesenchymal stem cells
UC-MSCs: Umbilical cord-derived mesenchymal stem cells
HGF: Hepatocyte growth factor
